# Modulation of Multiple Sclerosis and Its Animal Model Experimental Autoimmune Encephalomyelitis by Food and Gut Microbiota

**DOI:** 10.3389/fimmu.2017.01081

**Published:** 2017-09-05

**Authors:** Ward J. van den Hoogen, Jon D. Laman, Bert A. ’t Hart

**Affiliations:** ^1^Utrecht University, Utrecht, Netherlands; ^2^Department of Neuroscience, University Medical Center Groningen, University of Groningen, Groningen, Netherlands; ^3^Department of Immunobiology, Biomedical Primate Research Center, Rijswijk, Netherlands

**Keywords:** prebiotic, probiotic, autoimmunity, immunomodulation, fecal transplant, Food, gut microbiome

## Abstract

Multiple sclerosis (MS) is an autoimmune neurological disease characterized by chronic inflammation of the central nervous system (CNS), leading to demyelination, axonal damage, and symptoms such as fatigue and disability. Although the cause of MS is not known, the infiltration of peripherally activated immune cells into the CNS has a key pathogenic role. Accumulating evidence supports an important role of diet and gut microbiota in immune-mediated diseases. Preclinical as well as clinical studies suggest a role for gut microbiota and dietary components in MS. Here, we review these recent studies on gut microbiota and dietary interventions in MS and its animal model experimental autoimmune encephalomyelitis. We also propose directions for future research.

## Approach

PubMed was used as search engine for this review. Combinations of the following keywords were used: microbiota, microbiome, experimental autoimmune encephalomyelitis, multiple sclerosis, probiotic, prebiotic, synbiotic, fecal microbiota transplantation, and diet (see Box 1). In addition, preliminary reports acquired from conference abstracts have been used in the case of multiple sclerosis microbiota studies, to increase the amount of studies that our findings are based on. The studies of which only abstracts were available are noted in Table 3 as author, abstract. Since this article is not a systematic review, the 155 cited articles are a balanced selection out of approximately 200 papers. PubMed was last checked for new articles on July 10, 2017.

Box 1 **List of definitions used in this article.**

Microbiota: “’The population of microbes in a given anatomical niche in the human body (11).”

Microbiome: The collective genome of the microbiota (11).

Probiotic: “A live microbial feed supplement which beneficially affects the host animal by improving its microbial balance (154).”

Prebiotic: “Non-digestable food ingredients that beneficially affect the host by selectively stimulating the growth and/or activity of one or a limited number of bacterial species already resident in the colon, and thus attempt to improve host health (155).”

Synbiotic: “A mixture of probiotics and prebiotics that beneficially affects the host by improving the survival and implantation of live microbial dietary supplements in the gastrointestinal tract, by selectively stimulating the growth and/or by activating the metabolism of one or a limited number of health-promoting bacteria, and thus improving host welfare (155).”

## Introduction

Roughly 2.5 million people worldwide are affected by multiple sclerosis (MS), an autoimmune neurological disease of the central nervous system (CNS). Frequently observed symptoms are fatigue, numbness, loss of coordination, vision loss, dizziness, pain, cognitive defects, depression, and bladder and bowel dysfunction (1). MS can lead to serious motoric disability, as approximately 50% of patients require permanent use of a wheelchair 25 years after diagnosis (2). Although the cause of MS is not known, several lines of evidence point to a crucial pathogenic role of the immune system. Genome-wide association studies, neuropathological analyses, and successful therapy trials support the concept that peripheral interactions of environmental risk factors with MS-predisposing genetic factors elicit an autoimmune attack on the CNS causing the formation of lesions. Classically, lesions are defined as usually sharply edged demyelinated areas within the white matter with a variable degree of inflammation, axonal damage, and gliosis. The presence of immune cells (T and B cells, macrophages) and immune molecules (antibodies, complement) supports the characterization of MS as an autoimmune-mediated inflammatory disease.

A growing body of evidence indicates that gut microbiota can modify the incidence and/or course of immune-mediated, extraintestinal diseases (3). Since the gut microbiota are greatly impacted by diet, studies have also started to assess the contribution of gut microbiota and diet in MS and animal models of autoimmune demyelinating diseases such as experimental autoimmune encephalomyelitis (EAE). The aim of this review is to determine whether MS can be treated *via* modification of gut microbiota and dietary components. We describe key players of the immune system that are involved in the pathogenesis of MS, and we discuss how the cross talk of gut microbiota with the immune system can affect the expression of MS/EAE. We then review recent studies on gut emicrobiota and dietary components in the animal model of EAE and MS patients. We also review how gut microbiota can be modulated and propose future research topics.

## Key Players in MS Immunopathology

Multiple sclerosis presents itself in several forms of symptoms and disease courses. 85% of patients are diagnosed with relapsing remitting (RR) MS, which is characterized by alternating episodes of neurological symptoms (relapses) and recovery (remissions). During relapses, inflammatory active lesions can be detected in the CNS with imaging techniques, such as magnetic resonance imaging (MRI). As the disease progresses, recovery declines and 80% of RR MS patients develop secondary progressive (SP) MS within 10–20 years after initial diagnosis. SP MS does not come with periods of relapses and recovery, but increased disability gradually occurs along with axonal loss and decreased brain volume (atrophy). 10% of MS patients are diagnosed with primary progressive MS, which presents itself as SP MS directly from the start of the disease (2). The cause of MS is unknown, but various concepts have been proposed to explain disease etiology. An inside-out concept suggests that MS starts as a primary lesion in the CNS, for instance caused by an infection or by primary neurodegeneration inducing the release of self-antigens against which autoreactive T and B cells react. An outside-in concept, supported by animal research in EAE models, postulates that autoreactive T cells that have escaped thymic selection are activated by a peripheral infection. This activation can occur through antigens that closely resemble CNS antigens, bystander activation, novel autoantigen presentation, or recognition of sequestered CNS antigens. In both concepts, activated CD4^+^ T_h1_ and Th17 cells infiltrate into the CNS where they can be reactivated by resident antigen-presenting cells (APC), microglia for example. CD4^+^ T_h1_ and T_h17_ produce IFNγ and IL-17A, and the inflammatory reaction that follows increases the permeability of the blood–brain barrier and recruits other immune cells such as B cells and monocytes to the CNS. The inflammatory milieu also activates microglia, which in turn produce pro-inflammatory mediators which elicit demyelination and axonal loss (4). Naïve CD4^+^ T cells infiltrating the CNS can broaden the pattern of autoimmune reactions by epitope spreading, adding to the inflammatory milieu (2). CD8^+^ T cells likely also contribute to MS pathogenesis. They are found in high frequency in demyelinating lesions and correlate with axonal damage. Myelin-specific CD8^+^ T cells can be activated by epitope spreading, and up to a quarter of CD8^+^ T cells in active lesions are thought to be mucosa-associated invariant T (MAIT) cells. MAIT cells are able to produce IL-17 and are associated with the gut and liver (5). MS patients have increased serum levels of IL-18 (6), which activates MAIT cells and is inversely correlated with MAIT cell blood frequency (7). When costimulated by IL-18 and T-cell receptor stimulation, MAIT cells upregulate integrin very late antigen-4, which is involved in cell migration into the CNS (7). In addition, MAIT cells are depleted after efficacious autologous hematopoietic stem cell transplantation, a treatment which can be beneficial in MS patients (8). Therefore, MAIT cells are likely involved in the immunopathogenesis of MS. The relative significance of the different T cell subsets in human MS is not yet completely understood, as EAE is usually induced *via* complete Freund’s adjuvant (CFA), and interspecies immunological differences exist. Additionally, a more predominant CD4^+^ driven disease course is seen in EAE, while a more CD8^+^ T cell-driven immune response is seen in MS (2).

**Figure 1:**
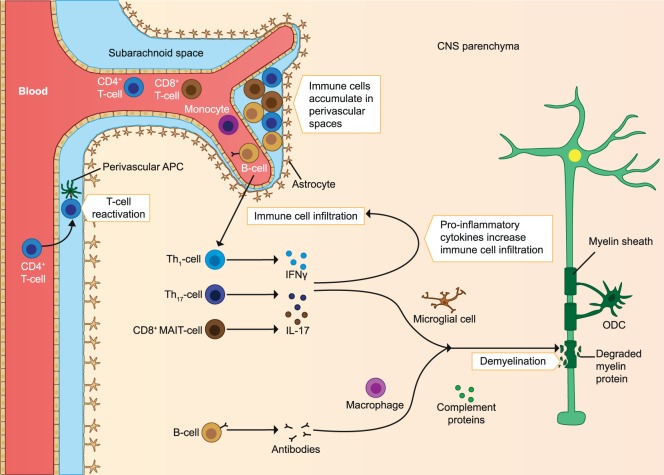
Immune cells involved in the pathology of early MS. Immune cells infiltrate the CNS and are reactivated by APC. The infiltrating T cells produce pro-inflammatory cytokines, which increases immune cell infiltration. The inflammatory milieu also activates microglia, which produce pro-inflammatory mediators and elicit demyelination and axonal loss. Autoantibodies produced by B cells cause damage to myelin through complement-mediated cytotoxicity and macrophage-mediated cytopathic reactions. As the disease progresses, immune cells accumulate in perivascular spaces. ODC, oligodendrocyte; MAIT, mucosa-associated invariant T cells; APC, antigen-presenting cells; CNS, central nervous system; MS, multiple sclerosis. The figure has been inspired by: Fugger et al., Grigoriadis et al., and Goverman (2, 4, 10).

**Figure 2:**
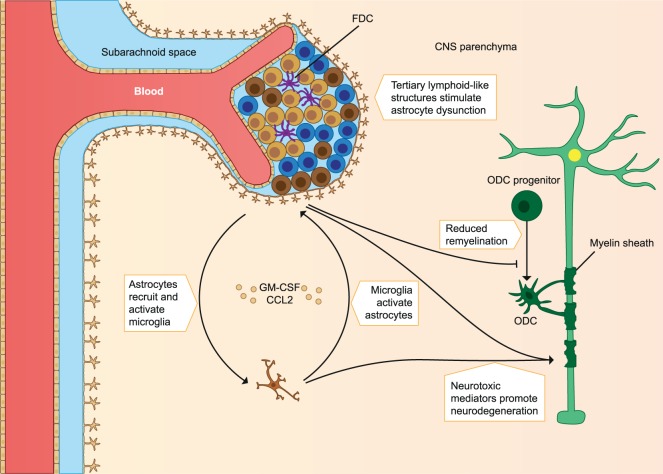
Immune cells involved in the pathology of late MS. Immune cell migration from the periphery into the CNS subsides, but chronic inflammation of the CNS still takes place. Chronic CNS inflammation is associated with tertiary lymphoid-like structures in perivascular spaces and dysfunctional astrocytes and microglia. Microglia activation promotes astrocyte production of CCL2 and GM-CSF, which recruits and activates more microglia. Astrocytes inhibit remyelination, and both microglia and astrocytes produce pro-inflammatory mediators that are neurotoxic and contribute to gradual neurodegeneration. FDC, follicular dendritic cells; ODC, oligodendrocyte; CNS, central nervous system; MS, multiple sclerosis. The figure has been inspired by: Fugger et al. and Goverman (2, 10).

In addition to T cells, autoreactive B cells may be activated in the periphery. B cells infiltrating the CNS locally produce autoantibodies, which bind myelin and cause damage to myelin *via* complement- and/or macrophage-mediated cytopathic reactions (CDC and ADCC). These B cells are also able to migrate out of the CNS and mature in the lymph nodes before migrating back to the CNS (9). Protective T_reg_ cells and their anti-inflammatory effects could also be defective. Protective cells include CD4^+^ Foxp3 expressing T_reg_, IL-10 producing T_r1_, and CD39^+^ T_reg_ cells. In MS patients, these cells are found in reduced frequency in the periphery and they have reduced immunosuppressive capacity compared to healthy individuals. Other regulatory cell types could also play a role, as they may increase after treatments. These include the CD8^+^ T_reg_ and IL-10 producing B_reg_ cells. In addition to defective regulatory cells, effector cells may escape their regulation when they are less sensitive to the suppression by T_reg_ cells (2). A clear imbalance of effector cells and regulatory cells is seen in early MS, which leads to a pro-inflammatory milieu in the CNS and promotes demyelination and axonal damage (see Figure 1) (2, 4, 10). In later stages of the disease immune cell migration from the periphery into the CNS subsides, but chronic CNS inflammation and neurodegeneration may continue to take place. This is associated with the formation of tertiary lymphoid-like structures within the CNS and associated meninges and with dysfunction of astrocytes and microglia. Microglial activation can promote dysfunction of astrocytes, after which astrocytes inhibit the maturation of myelin producing oligodendrocyte progenitors, resulting in reduced remyelination. Astrocytes can also produce CCL2 and GM-CSF, which further recruits and activates microglia and creates a self-sustaining feedback loop. Pro-inflammatory mediators such as reactive oxygen species (ROS) produced by astrocytes and microglia are neurotoxic, and the continuous CNS inflammation promotes gradual neurodegeneration (2) (see Figure 2).

## Key Players of the Human Gut Microbiota

**Figure 3:**
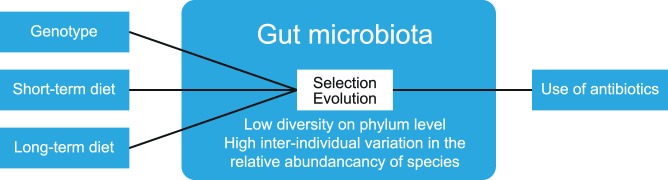
Factors that determine gut microbiota composition. The composition of gut microbiota is influenced by multiple factors, such as diet and host genotype. Within the gut, ecological processes such as selection and evolution take place. The use of antibiotics reduces the numbers and diversity of gut microbiota. The figure has been modified after: Walter and Donaldson et al. (14, 15).

The human gut serves as a host to many microbes (bacteria, archaea, viruses, and fungi). Newborn humans have a sterile gut, and colonization occurs through exposure to new flora depending on the mode of delivery, diet, and hygiene (11). Over the years, the gut microbiota increase in diversity and reach a maximum at adolescence, after which they remain fairly stable (11). The diversity of gut microbiota can be expressed as α-diversity, which shows the richness and distribution of taxa within one population. β-Diversity is used to measure differences between multiple populations, and it measures how many taxa are shared between populations (12). Gut microbiota display a low diversity at the phylum level, as bacteria from only 8 out of 55 phyla have been detected in the human gut (13). Yet, large interindividual variation exists in the relative abundance of microbiota members. This variation is not only greatly impacted by short-term dietary alteration but also influenced by long-term dietary habits, host genotype, and stochastic processes such as history of colonization and ecological processes, such as selection and evolution (14) (see Figure 3). Additionally, the use of antibiotics can reduce the number and diversity of gut microbiota, but these are largely restored to the pretreatment composition after a recovery period (15).

Studies investigating the human gut microbiota often use the sequencing of specific genes such as the 16s ribosomal RNA gene in stool samples to determine which species are present. This has to be interpreted with care, as the microbiota composition differs from the small intestine to the large intestine. The abundance of microbiota is increased along the gastrointestinal tract and while the jejunum is host to mainly aerobic species, the colon is dominated by anaerobic species. Sequencing of stool samples is therefore most informative of microbiota in the large intestine (16).

### Bacteria

The gut bacterial flora of healthy adults comprises mainly bacteria from the phyla Firmicutes and Bacteroidetes (16). These bacteria serve many functions. They aid in metabolism and nutrient availability by fermenting complex carbohydrates into short-chain fatty acids (SCFA), which can be used as an energy source by mucosa. Bacteria can also produce vitamins, such as vitamin K and components of certain vitamin B species (17), and play a role in the metabolism of medications. Gut microbiota also aid in the protection against pathogens by competitive exclusion (16, 17). The tremendous contribution of bacteria to host physiology is best seen in germfree (GF) mice, which have impaired development of the epithelium, musculature, and vasculature of the intestines as well as systemic defects, such as in immune functions and brain development. The gut-associated lymphoid tissue (GALT) is strongly underdeveloped in GF mice, with a reduced number of immune cells and lymph node size. These defects can be restored by colonization with bacteria, indicating the strong influence of bacteria on the host immune system. Conversely, the immune system promotes optimal growth and nutritional benefit and influences the species composition in the gut by the production of secretory IgA antibodies and bactericidal products, such as antimicrobial peptides (defensins and cathelicidins) (18). In addition, epithelial cells are covered by a mucus layer that is continuously consumed by bacteria and renewed by goblet cells (19). This mucus layer also contains peptides which aggregate bacteria but are not bactericidal and functions to keep bacteria at a safe distance from epithelial cells (20). A fiber-deprived diet increases the number and activity of mucus-degrading bacteria, thus reducing the mucus layer and increasing susceptibility to pathogens (21), indicating the importance and interplay of dietary components with microbiota and the immune system.

### Archaea

The majority of archaea found thus far in the human body are methanoarchaea, which all produce methane in the absence of oxygen. Because methods to efficiently detect archaea have only recently been developed, insight into the functional role of archaea in the gut is limited. Archaea seem to have a nutritional role as they form syntrophic interactions with bacteria and can favor the growth of fermenting bacteria (22). Archaea may also have immunogenic roles as lipids from *Methanobrevibacter* have powerful adjuvant properties (23) and exposure of monocyte-derived dendritic cells (DC) to archaea strains activates these cells (24).

### Viruses

Most studies on viruses in the human gut microbiota focus on DNA viruses, as over 95% of RNA viruses in the human gut are of plant origin and may have little influence on the gut microbiota of human hosts. Among DNA viruses, double-stranded DNA viruses from the order Caudovirales (Podoviridae, Siphoviridae, and Myoviridae) or single-stranded DNA viruses from the family Microviridae (25) have been identified in the human gut. Viruses can prevent infection of the host epithelium by binding to mucin glycoproteins, thereby limiting bacterial–epithelium adhesion. Additionally, viruses (phages) infect bacteria and exert effects on the host through the modulation of bacteria in the gut. Phages use distinct surface molecules for infecting bacteria, and therefore, have a tropism for specific strains of bacteria. After infection, phages can display a lysogenic phase, in which the phage integrates into the viral genome and stays there in a latent phase, the phage is then called a called prophage. Alternatively, phages can kill bacteria after viral replication which is called the lytic phase. Most gut bacteria are believed to have at least one prophage latently incorporated in their genome, and prophages can enter the lytic phase from a lysogenic phase after exposure to a range of stimuli (13, 25).

Bacteria protect themselves from virus infections by clustered regularly interspaced short palindromic repeats (CRISPR) in their genome. These are short DNA regions that contain foreign virus DNA in between them. When phages infect bacteria, bacteria transcribe the foreign DNA in the CRISPR region, which guides the bacteria to cleave the intruding phage and fight off the infection. During the battle between bacteria and phages, phages may incorporate whole gene segments from previously infected bacteria into bacterial chromosomes. This may disrupt host bacteria genes. Alternatively, the incorporated gene segment may increase bacterial fitness, thereby promoting colonization. Viruses can also introduce toxin-encoding genes into bacteria, which may promote dysbiosis of gut microbiota. Bacteria may use their prophages to their advantage, by producing them for lysis of competitor bacterial species. Finally, viruses can protect the host from overgrowth of dominant bacterial species, by killing bacteria when a high density is reached (13).

## The Influence of Gut Microbiota on Extraintestinal Tissues

In addition, gut microbiota may influence distant host tissues such as the CNS. Accumulating evidence indicates that gut microbiota affect various behaviors such as social interaction (26), nociceptive responses (27), depression (28), stress responsiveness, and anxiety (29). Gut microbiota also influence hippocampal neurogenesis (30), blood–brain barrier integrity (31), and microglia maturation (32).

Gut microbiota affect the CNS through multiple mechanisms. The gut enteric nervous system controls the motility and homeostasis of the gut, which influences the gut microbiota composition (33). Conversely, gut microbiota influence the enteric nervous system, and the vagus nerve provides a direct communicatory link between the gut and the CNS. Gut microbiota can produce neurotransmitters or precursors such as tryptophan, and microbiota metabolites can also directly influence the brain. For instance, fermenting bacteria produce SCFA, which can translocate into the brain and inhibit deacetylases, resulting in epigenetic changes (32). In addition, bacterial RNA, DNA, and proteins are detected in the human brain (34). Fragments of bacterial cell walls, such as peptidoglycan (PGN) may translocate to the brain and possibly influence brain development and social interaction (35). Bacteria also produce microbial-associated molecular patterns, which can be recognized by the host immune system with pattern recognition receptors, such as Toll- or Nod-like receptors and influence gut physiology (32, 35). The GALT is located along the small and large intestines and functions as immune surveillance of the gut. Gut microbiota can elicit an immune response in the GALT (36), which can be pro-inflammatory or anti-inflammatory, depending on the involved microbiota (37). The pro-inflammatory response of segmented filamentous bacteria (SFB) is best characterized. Studies in mice show that SFB enhance antigen presentation by DC resulting in greatly increased numbers of pro-inflammatory intraepithelial lymphocytes (IEL), such as γδ T cells and CD8^+^ T cells. SFB also increase IgA^+^ B cells and Th17 cells (13, 36). Th17 cells produce the cytokines IL-17A, IL-17F, IL-21, and IL-22. IL-17A and IL-17F control bacterial and fungal infections through the recruitment of neutrophils and more Th17 cells and increased production of β-defensin by epithelial cells. IL-22 induces epithelial cell proliferation, survival, tissue repair, and increased expression of antimicrobial molecules (13). In addition, gut bacteria such as *Helicobacter hepaticus* elicit a pro-inflammatory response, marked by increased numbers of T_h17_ and Th1 cells and pro-inflammatory cytokines excretion (38). Th1 cells are instrumental in fighting intracellular bacteria and viruses, *via* the production of IFNγ (13).

Gut microbiota are potent activators of innate lymphoid cells (ILC). ILC arise from common lymphoid precursors and respond rapidly to cytokines produced by the epithelium. ILC can be divided into three groups based on molecular markers: type 1 which expresses T box transcription factor (TBX21 or T-bet) in T cells, type 2 which expresses GATA binding protein and type 3 which expresses retinoid-related orphan receptor (ROR)γt. Type 3 ILC produce IL-17 and IL-22, and IL-22 production can be induced by bacterial metabolites acting on the aryl hydrocarbon receptor (AHR) (17). The pro-inflammatory reactions caused by gut microbiota may contribute to MS pathogenesis *via* the activation of autoreactive T-cells (36). Conversely, gut microbiota may also elicit an anti-inflammatory response, characterized by the induction of CD4^+^ T_reg_ cells. T_reg_ cells produce TGF-β and IL-10, which decrease pro-inflammatory cytokine production and cell proliferation. Clostridia species promote production of TGF-β, which helps maintain an anti-inflammatory milieu and is involved in the induction of T_reg_ cells (13, 36). Additionally, Bacteroides species increase the production of anti-inflammatory cytokines and suppress IL-17 production (36).

Gut microbiota also affect invariant natural killer T cells (iNKT cells); GF mice have increased iNKT cell numbers in their colon. These are a pro-inflammatory subset of T cells that express the invariant T-cell receptor α, with which they detect lipid antigens presented by CD1d molecules. They can promote T_h1_ and T_h17_ responses by producing IFNγ, IL-2, IL17A and tumor necrosis factor (TNF) (13).

## The Influence of phages on the Immune System

The influence of phages on the immune system is likely mostly indirect, through modulation of bacteria by mechanisms mentioned earlier. Phages may also directly communicate with the immune system. Humans are constantly sensitized with phage antigens present in food. This induces a constant low level of phage-neutralizing antibodies in human serum. After systemic phage administration in animals, these neutralizing antibodies are produced at higher titers. In humans, administration of enterobacteria PhiX174 phages induced IgM antibodies, followed by IgG antibodies after a second exposure (39). The function of antibodies is clearance of phages as illustrated by the lower clearance in B cell-deficient mice. A reduction of macrophages, natural killer, or T cells does not influence phage clearance (40). Conversely, phages can influence T cell functions *in vitro*. Mycobacteriophages were found to suppress phytohemagglutinin-induced activation of T lymphocytes from guinea pigs in a dose-dependent matter (41). Moreover, purified T4 phages can inhibit human T cell proliferation *via* the CD3 T-cell receptor complex, while phage lysates of *Staphylococcus aureus* can induce T-cell proliferation (41). In addition, phages can affect host phagocytes. Phagocytosis and ROS production may limit bacterial densities in the gut. Excessive phagocyte activity may also lead to pathology. Preincubation of monocytes and neutrophils with T4 and F8 phages from *Pseudomonas aeruginosa* reduced *in vivo* phagocytosis of *Escherichia coli*. T4 phages also reduced the ROS production by phagocytes exposed to LPS and bacterial cells, although this effect may be phage strain-dependent, because staphylococcal A3/R-purified phage (lysates) did not affect ROS production (41). The effect of phages depends also on the type of preparation, as T4 lysates promote IL-6 production by monocytes but purified T4 phages do not (41). Most effects of phages are anti-inflammatory, as reflected by mitigated phagocytosis, ROS production, and lymphocyte proliferation. However, phage lysates of *S. aureus* are likely more pro-inflammatory. Overall, effects of phages on the host immune system are complex and depend on phage identity, phage tropism, and preparation.

**Figure 4:**
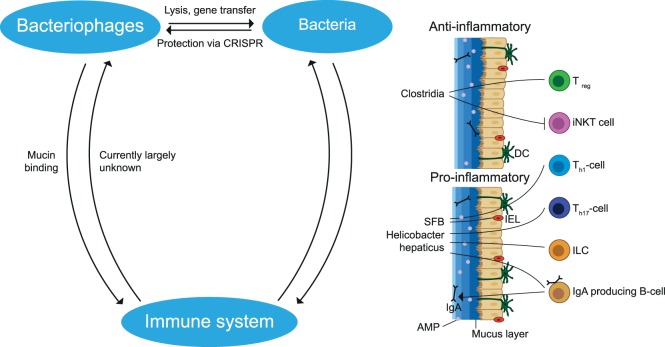
Interactions between members of the gut microbiota and the immune system. Bacteriophages can infect and lyse bacteria or undergo a lysogenic cycle in which they stay dormant inside bacteria. During this process, gene segments may be transmitted which influences the fitness of the bacteria. Bacteria protect themselves from phage infection by CRISPR. Bacteria may cause a pro- and anti-inflammatory effect dependent on the bacterial species. Anti-inflammatory effects include the induction of T_reg_ cells and the reduction of iNKT cells. Pro-inflammatory effects include induction of T_h1_, T_h17_, IgA producing B cells and stimulation of IL-22 production by ILC, which increases AMP production. These immune cells and the mucus layer protect the epithelial cells from being infected by bacteria. In addition, phages limit bacteria–epithelial adhesion by binding to the mucus layer. The effects of the gut immune system on phages remain largely unknown. SFB, segmented filamentous bacteria; AMP, antimicrobial peptides; iNKT, invariant natural killer T; ILC, innate lymphoid cells; IEL, intraepithelial lymphocytes; DC, dendritic cell; CRISPR, clustered regularly interspaced short palindromic repeats. The figure has been inspired by: Glenn and Mowry (13).

Collectively, variable exposure to the environment and food greatly influences gut microbiota composition. Elements of the gut microbiota constantly interact with each other and with the host and are essential for the normal development of the immune system and CNS. Pro- and anti-inflammatory effects have been attributed to different microbial species and an imbalance may contribute to autoimmune disease may contribute to autoimmune disease (see Figure 4).

## EAE Models

Due to the inaccessibility of the CNS of MS patients, animal models are used for translational research into the pathogenesis of MS and for therapy development. As in none of the available models the immunological and pathological complexity of MS is fully replicated, multiple models are being used. Depending on the research question models of MS-like demyelination are induced chemically (cuprizone, lysolecithin), with neurotropic viruses (Theiler’s murine encephalitis virus, Semliki Forest virus), through specific cytokine overexpression in the CNS, diphtheria toxin-based depletion of oligodendrocytes, and active immunization with myelin components in suitable adjuvants (42, 43). The active immunization model, known as EAE, is the most widely used preclinical MS model. EAE can be induced in a wide variety of laboratory animal species including mice, rats, rabbits, guinea pigs, and non-human primates by inoculation of myelin antigens formulated with a strong adjuvant, such as CFA. For synchronous EAE induction at high incidence in mice, the immunization is usually supplemented with injection of *Bordetella pertussis* toxin. Different myelin antigens and dosages are used to model the heterogeneity of MS. For example, SJL mice develop a RR type of EAE after sensitization against residues 79–87 of myelin basic protein (MBP_79–87_) or residues 131–151 of proteolipid protein (PLP_131–151_). C57Bl/6 mice can develop RR EAE after immunization with a low dose of myelin oligodendrocyte glycoprotein residues 35–55 (MOG_35–55_) and develop chronic EAE without remission upon immunization with a high dose of MOG_35–55_ (44). It is increasingly felt that the usage of strong adjuvants precludes a role of subtle regulatory mechanisms. For such studies, passive EAE models induced by the transfer of activated T cells from a donor with actively induced EAE or spontaneous EAE models established in mice expressing transgenic T and/or B cell receptors specific for myelin antigens may be more useful (43).

Although EAE models have proven their relevance as a preclinical test system for new therapeutics they also have their limitations. The highly artificial way by which the autoimmune process is activated in CFA-based EAE models does not necessarily reflect the natural immune response toward self-antigens. As an illustration, the EAE model is dominated by CD4^+^ T cells, whereas CD8^+^ T cells are likely more immunodominant in MS patients (44). Another difference between EAE and MS is that mice with EAE mostly show lesions in the spinal cord, whereas MS patients mostly show lesions in the brain (43). A noticeable exception is formed by the atypical EAE models in marmosets, a small-bodied Neotropical primate, which more closely approximate MS with respect to clinical, pathological, and immunological presentation (45).

## Gut Microbiota-Based Interventions in EAE Models

The first evidence that gut microbiota are involved in the pathogenesis of EAE stems from decades ago. Already in 1993, it was discovered that transgenic mice expressing T-cell receptors specific for MBP fail to develop EAE when they were housed in a sterile, specific pathogen-free (SPF) environment, while mice housed in a non-sterile environment did develop EAE (46). To determine the role of gut microbiota in EAE, GF models, antibiotic treatments, probiotic mixtures, bacterial products, and diet-based interventions have been used. These studies are summarized in Tables 1 and 2.

**Table 1:** The role of gut microbiota in EAE.

Reference	Animal model	Intervention	Clinical score	Immune response
Berer et al. (47)	SJL anti-MOG_92–106_ TCR^tg^	Germfree housing	Protected	Reduced T_h17_, impaired B-cell recruitment to brain-draining lymph nodes Reduced anti-MOG B cell response

Lee et al. (48)	C57Bl/6 MOG_35–55_	Germfree housing	Decreased	Reduced T_h1_ and T_h17_, increased T_reg_ Reduced DC capacity to induce T_h1_ and T_h17_ responses

Yokote et al. (49)	C57Bl/6 MOG_35–55_	Broad spectrum antibiotics	Decreased	Decreased pro-inflammatory cytokines, decreased T_h17_

Ochoa-Repáraz et al. (50)	SJL PLP_139–151_; C57Bl/6 MOG_35–55_	Broad spectrum antibiotics	Decreased	Reduced pro-inflammatory cytokines, increased T_reg_ Increased CD11c^high^CD103^+^ cells

Ochoa-Repáraz et al. (51)	C57Bl/6 MOG_35–55_	Broad spectrum antibiotics	Decreased	Increased IL-10 producing CD5^+^ B-cells Shift from T_h1_ and T_h17_ toward T_h2_ response

Ochoa-Repáraz et al. (52)	SJL PLP_139–151_	Oral administration of *Bacteroides fragilis*	Decreased	Increased T_reg_, reduced T_h17_

Ochoa-Repáraz et al. (52)	SJL PLP_139–151_	Oral administration of PSA^−/−^ *B. fragilis*	Normal	Normal

Ezendam et al. (53)	Lewis rats MBP	Oral administration of *Bifidobacterium animalis*	Decreased duration	Not investigated

Lavasani et al. (54)	C57Bl/6 MOG_35–55_	Oral administration of three *Lactobacilli* strains	Decreased	Reduced T_h1_ and T_h17_, increased T_reg_, IL-10 dependent

Takata et al. (55)	C57Bl/6 MOG_35–55_; SJL PLP_139–151_	Oral treatment with heat-killed *Pediococcus acidilactici*	Decreased	Reduced T_h1_ and T_h17_, increased T_reg_

Maassen and Claassen (56)	Lewis rats MBP; SJL PLP_139–151_	Oral treatment with commercially available probiotic drinks containing *Lactobacillus casei*	Decreased in Lewis rats, no effect in SJL model	Not investigated

Kwon et al. (57)	C57Bl/6 MOG_35–55_	Oral administration of *Bifidobacterium bifidum, Streptococcus thermophilus* and three *Lactobacillus* strains	Decreased	Reduced T_h1_ and T_h17_ response, increased T_reg_

Rezende et al. (58)	C57Bl/6 MOG_35–55_	Oral administration of recombinant HSP65-producing *Lactococcus lactis*^tg^	Decreased	Decreased IL-17, increased IL-10, dependent on increased CD4^+^LAP^+^ T_reg_

Wang et al.; Ochoa-Repáraz et al. (59–61)	SJL PLP_139–151_; C57Bl/6 MOG_35–55_	Oral treatment with *B. fragilis*-produced PSA	Decreased	Reduced T_h1_ and T_h17_, increased T_reg_ and CD103^+^ DC, increased CD39^+^ T_reg_

Kadowaki et al. (62)	2D2 anti-MOG TCR^tg^; C57Bl/6 MOG_35–55_	Adoptive transfer of CD4^+^ induced IEL	Decreased	Reduced T_h17_ CD4^+^ induced IEL are dependent on gut microbiota and diet

Maassen et al. (63)	Lewis rats, MBP_72–85_	Oral administration of live/intranasal administration of soluble cell extracts from myelin proteins producing *L. casei*	Decreased, extracts from guinea pig MBP producing bacteria increased	Not investigated

*Germfree housing, antibiotics, probiotics, and bacterial products affect the EAE clinical score*.

*DC, dendritic cells; EAE, experimental autoimmune encephalomyelitis; IEL, intraepithelial lymphocytes; MBP, myelin basic protein; MOG, myelin oligodendrocyte glycoprotein; PLP, proteolipid protein; PSA, polysaccharide A*.

**Table 2:** Dietary interventions in EAE.

Reference	Animal model	Intervention	Clinical score	Immune response
Haghikia et al. (64)	C57Bl/6 MOG_35–55_	Oral administration of propionic acid (short-chain fatty acid) and lauric acid (long-chain fatty acid)	Decreased	Increased T_reg_, reduced T_h17_ on PA treatment Increased T_h1_ and T_h17_, decreased *Prevotellaceae* and *Bacteroidetes* on lauric acid treatment

Lemire and Archer (65)	SJL spinal cord homogenate	Intraperitoneal vitamin D administration	Decreased	Reduced antibodies against MBP

Cantorna et al. (66)	B10.PL MBP_79–87_	Dietary vitamin D supplementation	Decreased	Not investigated

Spach et al. (67)	C57Bl/6 MOG_35–55_	Dietary vitamin D supplementation	Decreased	Reduced inflammatory cells, IFNγ in the spinal cord, IL-10 dependent

Piccio et al. (68)	SJL PLP_139–151_; C57Bl/6 MOG_35–55_	40% caloric restriction	Decreased	Increased plasma levels of corticosterone, adiponectin, reduced plasma levels of IL-6 and leptin

Esquifino et al. (69)	Lewis rats spinal cord homogenate	33 and 66% caloric restriction	66% caloric restriction protected from EAE signs	Reduced splenic CD8^+^ T cells and B cells, reduced lymphoid and thymic CD4^+^ T cells and B cells and IFNγ production

Kafami et al. (70)	C57Bl/6 MOG_35–55_	Intermittent feeding	Decreased	Not investigated

Harbige et al. (71)	SJL MOG_92–106_	Oral γ-linolenic acid treatment	Decreased	Increased TGF-β, prostaglandin E_2_ production by spleen mononuclear cells

Harbige et al. (72)	Lewis rats, guinea pig spinal cord homogenate	Oral γ-linolenic acid treatment	Decreased	Not investigated

Kong et al. (73)	C57Bl/6 MOG_35–55_	DHA-rich diet	Decreased	Reduced T_h1_ and Th17 cell differentiation, reduced amounts of T_h1_, T_h17_ found in the spleen and spinal cord of mice on a DHA-rich diet. *In vitro*, DHA reduced the expression of costimulatory molecules on DC and reduced their production of pro-inflammatory cytokines

Unoda et al. (74)	C57Bl/6 MOG_35–55_	EPA supplementation	Decreased	Increased expression of PPAR α, β, and γ on CD4^+^ T cells in the spinal cord, reduced IFNγ and IL-17 cytokine production. CD4^+^ T cells from the spleen of EPA-treated mice expressed increased mRNA levels of Foxp3, but also of IL-17 and RORγt

Salvati et al. (75)	Dark agouti rats, guinea pig spinal cord homogenate	EPA supplementation	Delayed time before EAE symptoms appeared	Increased myelination of axons in the spinal cord

Kim et al. (76)	C57Bl/6 MOG_35–55_	Ketogenic diet	Decreased	Reduced T_h1_, T_h17_, and pro-inflammatory cytokines

Choi et al. (77)	C57Bl/6 MOG_35–55_	Cycles of fasting Ketogenic diet	Decreased	Fasting increased T_reg_, corticosterone, reduced CD11^+^ DC, T_h1_, T_h17_, pro-inflammatory cytokines Ketogenic diet: not investigated

Jörg et al. (78)	C57Bl/6 MOG_35–55_	High-salt diet	Increased	Increased T_h17_

Krementsov et al. (79)	C57Bl/6 MOG_35–55_, SJL PLP_135–151_	High-salt diet	Increased in C57Bl/6 mice, in SJL only increased in females	No difference in T_reg_, T_h1_, and Th17 cells

Wu et al. (80)	C57Bl/6 MOG_35–55_	High-salt diet	Increased	Increased T_h17_ in CNS and mesenteric lymph nodes, SGK-1 signaling dependent

Kleinewietfeld et al. (81)	C57Bl/6 MOG_35–55_	High-salt diet	Increased	Increased inflammatory cell infiltration into the CNS, increased T_h17_

Veldhoen et al. (82)	C57Bl/6 MOG_35–55_	FICZ administration	Increased	Increased IL-17- and IL-22-producing CD4^+^ T cells in the spinal cords

Quintana et al. (83)	C57Bl/6 MOG_35–55_	FICZ, ITE, TCDD administration	FICZ increased, ITE and TCDD reduced	FICZ: increased IL-17^+^CD4^+^ and IFNγ^+^CD4^+^ T cells in the spleen TCDD: increased Foxp3^+^ T_reg_

Rothhammer et al. (84)	C57Bl/6 MOG_35–55_	Tryptophan-deficient diet, supplementation with tryptophan metabolites and tryptophanase	Increased, supplementation reduced EAE scores	IFN-1 signaling induces AHR expression in astrocytes, supplementation does not reduce EAE scores in astrocyte-specific AHR knockout mice

Stoye et al. (85)	SJL PLP_139–151_	Intraperitoneal injection of ZnAsp	Decreased, but increased on high doses	Reduced proliferation of stimulated human T-cells, reduced pro-inflammatory cytokine production

Schubert et al. (86)	SJL PLP_139–151_	Oral ZnAsp supplementation	Decreased, but increased on high doses	Reduced proliferation of stimulated human T-cells, reduced pro-inflammatory cytokine production

Kitabayashi et al. (87)	C57Bl/6 MOG_35–55_	Oral Zink supplementation	Decreased	Not investigated

Rosenkranz et al. (88)	C57Bl/6 MOG_35–55_	Intraperitoneal injection of ZnAsp	Decreased	Reduced systemic Th17 cells and increased Foxp3^+^ T-cells in the spinal cord

Scelsi et al. (89)	Guinea pigs, spinal cord homogenate	Oral selenium supplementation	Increased on high doses, normal on normal doses	Not investigated

Chanaday et al. (90)	Wistar rats, whole MBP	Intraperitoneal and oral diphenyl diselenide	Diphenyl diselenide was toxic when intraperitoneally administered, reduced when orally administered	Reduced number of macrophages in the CNS, reduced MBP-specific T-cell proliferation

Xue et al. (91)	C57Bl/6 MOG_35–55_	Intraperitoneal tocopherol administration	Decreased	Reduced MOG-specific splenocyte proliferation. Splenocytes incubated with tocopherol produced less IFNγ

Blanchard et al. (92)	C57Bl/6 MOG_35–55_	Intraperitoneal TFA-12 administration	Decreased	Reduced inflammation of the CNS, astrogliosis and demyelination. Induces oligodendrocyte maturation

Racke et al. (93)	SJL, MOG-incubated lymph node cells	Dietary 13-cis-retinoic acid and 4-HPR	Decreased	Not investigated

Zhan et al. (94)	C57Bl/6 MOG_35–55_	Intraperitoneal all-trans retinoic acid administration	Decreased	Reduced DC maturation, reduced pro-inflammatory monocytes in the subarachnoid space. Reduced numbers of T_h1_ and Th17 cells in the draining lymph nodes

Xiao et al. (95)	C57Bl/6 MOG_35–55_	Intraperitoneal all-trans retinoic acid administration	Decreased	Inhibits T_h17_ differentiation by reducing expression of the IL-6 and IL-23 receptor

*Dietary interventions affect the microbiota and EAE clinical scores*.

*AHR, aryl hydrocarbon receptor; CNS, central nervous system; DC, dendritic cells; DHA, docosahexaenoic acid; EAE, experimental autoimmune encephalomyelitis; EPA, eicosapentaenoic acid; FICZ, 6-formylindolo[3-2b]carbazole; ITE, 2-(1′H-indole-3′carbonyl)-thiazole-4-carboxylic acid methyl ester; MBP, myelin basic protein; MOG, myelin oligodendrocyte glycoprotein; PLP, proteolipid protein; SGK-1, serum/glucocorticoid kinase 1; TCDD, 2,3,7,8-tetrachlorodibenzo-p-dioxin*.

### GF Models

Germfree mice, which are bred and raised in a sterile environment, display significantly attenuated disease in both spontaneous and actively induced EAE models (47, 48). In the actively induced EAE model, GF mice develop EAE at a reduced incidence, while in mice developing overt disease, symptoms are milder and of shorter duration compared to conventionally colonized mice. This has been attributed to reduced mesenteric lymph node DC capacity to induce T_h1_ and T_h17_ responses. GF mice have reduced IL-17 and IFNγ producing CD4^+^ T cells in their spinal cord. This is accompanied by increased CD4^+^CD25^+^Foxp3^+^ T_reg_ cells in draining lymph nodes and spleen. Compared to GF mice, monocolonization with SFB significantly increases EAE clinical scores, with increased IL-17 and IFNγ production in the small intestines and spinal cord (48). SPF-bred MOG TCR transgenic mice develop spontaneous EAE at high incidence, but appear completely EAE resistant under GF conditions (47). Upon colonization with conventional microbiota, EAE quickly developed (47).

Compared to conventionally colonized mice, GF mice have reduced IL-17 producing CD4^+^ cells in Peyer’s patches and lamina propria, but no difference is seen in mesenteric lymph nodes or other remote organs. Additionally, MOG-immunized GF mice had reduced autoreactive anti-MOG antibodies which could be increased by colonization. It is thought that MOG-specific B cells are activated and recruited into deep cervical lymph nodes by helper T cells. Once there, B cells encounter MOG imported from the lymphatic vessels and undergo proliferation, immunoglobulin class switching, and somatic hypermutation (47).

Germfree mice also have reduced gut luminal extracellular adenosine 5′-triphosphate (ATP). ATP can be derived from bacteria, and ATP activates lamina propria CD70^high^CD11c^low^ DC, leading to the production of IL-6 and IL-23 which are important for the differentiation of Th17 cells. Systemic and rectal administration of ATP increases the number of Th17 cells in GF mice. Although investigated in a colitis model, administration of ATP increases T_h17_ response (96). This may be relevant for EAE due to the role of Th17 cells in EAE. Administration of alkaline phosphatase, which can neutralize ATP and LPS, reduced the clinical signs of EAE when given presymptomatically but not in the acute or chronic phase (97). Besides reduced gut luminal ATP, GF animals have reduced tight junctions and increased permeability at the blood–brain barrier, potentially affecting EAE (32).

A general caveat in the usage of GF mice for the modeling of human autoimmune disease is that the immunocompetence of such mice is seriously disturbed. Even the frequently used SPF-bred mice are immunologically incomparable to adult humans. SPF-bred mice have reduced cervix mucosal memory T cells, and a relatively lower number of differentiated effector memory CD8^+^ T-cells in their blood, a signature which is comparable to neonatal humans. Feral and pet store mice are immunologically more comparable to adult humans, and SPF-bred mice could be immunologically normalized by cohousing them with pet store mice (98). This indicates that exposure to pathogens affects the immune system and that studies using GF and SPF animals may not always translate well into the clinic.

### Antibiotics

Antibiotic treatments modulate gut microbiota and this also affects EAE. An oral cocktail of non-absorbing antibiotics [kanamycin, colistin, and vancomycin (KCV)] administered 1 week before active EAE induction reduces disease scores. The impairment of normal EAE development is accompanied by a reduction of IFNγ, TNFα, IL-6, and IL-17 production by MOG-reactive T cells from draining lymph nodes. KCV also reduced the number of Th17 cells in mesenteric lymph nodes while this effect was not seen in iNKT cell-deficient mice, indicating that iNKT cells are mechanistically important in KCV treatment. Since no differences were found in mesenteric lymph node iNKT cells, Foxp3^+^ T_reg_ cells and T_h17_ promoting cytokines, KCV treatment likely exerts its effect upstream of the mesenteric lymph nodes. In the KCV-treated group, reduced expression of IL-21 and IL-23 cytokines were found in lamina propria lymphocytes, and it is therefore speculated that KCV alters iNKT cells in the lamina propria, ultimately leading to Th17 cells in the mesenteric lymph nodes (49).

With a different antibiotics mixture (ampicillin, vancomycin, neomycin sulfate, and metronidazole) orally administered before EAE induction, normal EAE development was also impaired. This has been attributed to an increase in Foxp3^+^ Treg cells in the mesenteric and cervical lymph nodes (deep or superficial lymph nodes not specified) of antibiotic-treated mice. The increase in Foxp3^+^ Treg cells is likely a result of increased CD11c^high^CD103^+^ DC in Peyer’s patches and mesenteric lymph nodes. CD11c^high^CD103^+^ DC enhance the conversion of naive CD4^+^ T cells into Foxp3^+^ Treg cells (50). In addition, this antibiotics mixture increases IL-10 producing CD5^+^ B cells in cervical lymph nodes (deep/superficial lymph nodes not specified). Adoptive transfer of splenic CD5^+^ B cells obtained from mice treated with antibiotics into naïve recipient mice which were MOG_35–55-_immunized 1 day post transfer reduced EAE disease score. The reduced disease score was associated with a shift from a T_h1_/T_h17_ cytokine profile toward a Th2 cytokine profile (51). Thus, this antibiotic treatment induced both regulatory T and B cells which protected against EAE.

### Probiotics

The finding that gut microbiota can elicit pro- and anti-inflammatory reactions, has raised the interest for treatment of EAE with bacteria. Prophylactic treatment with *Bifidobacterium animalis* decreases the duration of EAE symptoms (53) and three commercially available probiotic drinks containing strains of *Lactobacillus casei* could reduce the EAE disease score in Lewis rats. However, no significant effects of these drinks have been observed in an SJL mouse model (56).

Prophylactic use of *Lactobacilli* monostrains reduces autoreactive T cells and prevents EAE. However, using these monostrains, established EAE could not be reversed. When three *Lactobacilli* strains are combined in a mixture, *Lactobacilli* therapeutically suppress disease progression and reduce clinical signs in MOG_35–55_-immunized mice. The beneficial effect was IL-10 dependent, and treatment induced CD4^+^CD25^+^ T_reg_ cells in the mesenteric lymph nodes. The treatment also reduced T_h1_ and T_h17_ cytokines and increased IL-10 production in cultures of splenocytes cultured with autoantigen. In addition, reduced IL-17 and increased IL-10 levels were found in the CNS of treated animals (54).

Prophylactic treatment with heat-killed *Pediococcus acidilactici* resulted in reduced EAE scores and decreased MOG_35–55_-induced IL-17 and IFNγ production from draining lymph node cells and splenocytes. Treatment also increased CD4^+^ IL-10^+^ cells in mesenteric lymph nodes and the spleen but not in the lamina propria. It is therefore thought that the treatment with heat-killed *P. acidilactici* activates inhibitory DC, which then migrate to the mesenteric lymph nodes to locally induce IL-10^+^ T_reg_ cells. Treatment with heat-killed *P. acidilactici* also reduced the disease score in established EAE (55).

Prophylactic treatment with a cocktail of *Bifidobacterium bifidum, Streptococcus thermophilus*, and three *Lactobacillus* subspecies suppressed the EAE incidence and severity in MOG_35–55_-immunized C57Bl/6 mice. The beneficial effect was associated with reduced T_h1_ and Th17 cell frequency and concomitant cytokine production along with increased IL-10 production in lymph nodes and spinal cord. IL-10 production by CD4^+^ T cells and CD11c^+^ DC is also increased in the spinal cord. When given after EAE immunization, this cocktail delays disease onset but cannot halt disease progression (57).

Intraepithelial lymphocytes are located in the epithelial layers of mucosal linings, e.g., of gastrointestinal and reproductive tracts. They comprise CD2^−^CD5^−^ natural, and CD2^+^CD5^+^ induced T cells, which release cytokines upon antigenic stimulation without the need of antecedent priming. Natural IEL acquire their activated phenotype in the presence of self-antigens in the thymus, while induced IEL acquire their phenotype in post-thymic cognate interaction with antigen. Adoptive transfer of CD4^+^ IEL prior to EAE induction results in reduced disease severity. IEL cells transferred to the CNS upregulate LAG-3, CTLA-4, and TGF-β, but it is still unclear where and how they acquire their phenotype. It is evident, however, that IEL are affected by gut microbiota and can be induced by dietary components, such as aryl hydrocarbon ligands. It has not been tested whether CD4^+^ IEL can reduce established EAE (62).

A series of observations shows the therapeutic value of polysaccharide A (PSA), produced by the bacterium *Bacteroides fragilis*. First, mice treated with antibiotics and recolonized with *B. fragilis* have reduced EAE disease scores compared to those not recolonized. Second, mice recolonized with PSA-deficient *B. fragilis* develop a normal EAE disease course (52), while mice colonized with wild-type *B. fragilis* have a milder disease course. The clinical effect is mirrored by reduced Th17 cells in the periphery and reduced IL-17 but increased IL-10 levels in the brains. Wild-type *B. fragilis* recolonized mice also have increased CD103^+^ DC in their cervical lymph nodes (deep/superficial lymph nodes not specified). CD103^+^ DC are known to convert naïve CD4^+^ T cells into IL-10 producing Foxp3^+^ T cells (52).

### Bacterial Products

Oral treatment with purified PSA protects against EAE both in a prophylactic and therapeutic mode. PSA treatment led to increased CD103^+^ DC in the cervical lymph nodes (deep/superficial lymph nodes not specified) (59). Additionally, PSA is also recognized by DC near mesenteric lymph nodes in a toll-like receptor 2-dependent mechanism. PSA-exposed DC migrate to the mesenteric lymph nodes where they induce IL-10 producing T_reg_ cells (60, 99). A type of T_reg_ cell specifically induced by PSA has surface expression of ectonucleoside triphosphate diphosphohydrolase-1 (NTPDase-1; CD39). The NTPDase-1 converts extracellular pro-inflammatory ATP into 5′AMP, which can be further degraded to adenosine which, in contrast to ATP, has anti-inflammatory properties exerted *via* adenosine receptors (60, 99). CD39^+^ T_reg_ cells have increased migratory capacity and are more abundant in the CNS of PSA-treated mice. Upon adoptive transfer, CD39^+^ T_reg_ cells are protective against EAE in MOG-induced C57Bl/6 mice. PSA does not protect against EAE in CD39-deficient mice (61). Multiple bacteria species present in the large intestine are able to produce SCFA (acetate, propionate, and butyrate) by fermentation of dietary fibers. Through its effect on histone deacetylases butyrate can induce epigenetic modifications, such as acetylation of the Foxp3 locus. Butyrate can also stimulate DC and macrophages to secrete IL-10 and retinoic acid through G-protein-coupled receptors, such as Gpr41, 43, and 109a. SCFA may also act on epithelial cells, e.g., by stimulating TGF-β production. The acetylation of the Foxp3 locus as well as production of IL-10, retinoic acid, and TGF-β facilitates differentiation of naïve CD4^+^ T cells into anti-inflammatory Foxp3^+^ T cells (100).

### Modified Probiotics

Bacteria can also be used as vector to deliver proteins into the gut and induce tolerance against these proteins. Oral pretreatment with recombinant *L. casei* which produce myelin antigens can reduce EAE scores in Lewis rats. In addition, intranasal pretreatment with soluble cell extracts of bacteria producing MBP_72–85_ could also reduce the EAE disease score, while extracts of bacteria producing guinea pig MBP exacerbated the EAE disease score (63). Effects of *Lactobacilli* on the immune system are strain dependent, as different *Lactobacilli* induce distinct cytokine profiles in the mucosa (101). In addition, the growth phase (log vs stationary) of the bacterial culture is of importance, since this influences the IgG1/IgG2a antibody subclass ratio, which is indicative of the T_h2_/T_h1_ pathway ratio (102). The differential effect of *Lactobacilli* strains and the influence of the phase of the bacterial culture should be taken into account when designing probiotic preparations.

Pretreatment with *Lactococcus lactis*, expressing heat shock protein 65 as a transgene, suppressed EAE development in MOG_35–55_-immunized C57Bl/6 mice. The clinical effect was associated with reduced MOG-induced IL-17 production by splenocytes and increased IL-10 production by MOG-stimulated mesenteric lymph node cells. The treatment also caused increased T_reg_ cells in the spleen, inguinal and mesenteric lymph nodes, and spinal cord. *In vivo* depletion of CD4^+^LAP^+^ T_reg_ cells abrogated the protective effect of transgenic *L. lactis*, indicating that the increased T_reg_ cells are mechanistically important. The efficacy of this treatment during established EAE has not yet been investigated (58).

## Diet-Based Interventions in EAE Models

The notions that SCFA (<6 carbons) produced by gut microbiota modify immune functions and that SCFA are consumed through the diet underlie the hypothesis that the diet influences microbiota, the immune system, and ultimately EAE. Prophylactic oral treatment with propionic acid, a SCFA that is ingested with food, increases T_reg_ cells and reduces the EAE disease score. In contrast, long-chain fatty acids (LCFA; 13–21 carbons) increase EAE scores, which is associated with increased T_h1_ and Th17 cells (64). Of note, SCFA can migrate into the CNS and serve as fuel for CNS neurons and glial cells. The microbiota composition of these mice also differs, as those fed with lauric acid, a saturated LCFA, have reduced *Prevotellaceae* and *Bacteroidetes* in their gut microbiota. This illustrates the impact that dietary components can have on the immune system and EAE expression, possibly through the modification of gut microbiota. Fatty acids can also have beneficial effects on EAE, regardless of their length, when they are unsaturated. These lipids have one or more double bonds between the carbon atoms of their hydrocarbon chain. Fatty acids with a double bond at the third carbon atom counted from the methyl (–CH3) tail of the chain are Ω-3 fatty acids and with a double bond at the sixth carbon atom are Ω-6 fatty acids. Oral treatment with the Ω-6 fatty acid γ-linolenic acid reduced the EAE clinical scores in MOG_92–106_-immunized SJL mice. This was associated with increased TGF-β, prostaglandin E_2_ production by spleen mononuclear cells (71). Oral treatment with oils containing γ-linolenic acid also reduced the EAE clinical scores in Lewis rats, where EAE was induced with guinea pig CNS matter homogenate (72). Ω-3 fatty acids can also reduce the EAE clinical scores. A diet rich in docosahexaenoic acid (DHA) starting 5 weeks before induction of EAE with MOG_35–55_ reduced the EAE clinical scores in C57Bl/6 mice. This was found associated with reduced T_h1_ and Th17 cell differentiation, and reduced amounts of these cells were found in the spleen and spinal cord of mice on a DHA-rich diet. *In vitro*, DHA reduced the expression of costimulatory molecules on DC and reduced their production of pro-inflammatory cytokines (73). A diet with the triglyceride form of DHA, starting before EAE induction also reduced the EAE clinical scores in the same mouse model. *In vitro*, pretreatment of microglia cells with the triglyceride form of DHA reduced microglial oxidative stress and production of nitric oxide and inflammatory cytokines (103). Eicosapentaenoic acid (EPA) supplementation starting 7 days after EAE induction reduced the EAE clinical scores in MOG_35–55_-immunized C57Bl/6 mice. EPA is a ligand for PPAR α, β, and γ and increased their expression on CD4^+^ T cells in the spinal cord, while reducing IFNγ and IL-17 cytokine production. CD4^+^ T cells from the spleen of EPA-treated mice expressed increased mRNA levels of Foxp3, but also of IL-17 and RORγt (74). When an EPA rich diet was given at the time point of EAE induction, it delayed the time before EAE symptoms appeared in guinea pig spinal cord homogenate-immunized dark agouti rats. This was associated with increased myelination of axons in the spinal cord (75). A ketogenic diet, characterized by a high fat to protein and carbohydrate diet, also reduced the EAE clinical score when given before EAE induction. This was associated with reduced T_h1_ and Th17 cells in the CNS and lymph nodes (the authors did not specify which lymph nodes). Additionally, mice on a ketogenic diet had reduced levels of pro-inflammatory cytokines in their lymph nodes and CNS (76). Another study confirmed the beneficial effect of the same ketogenic diet but did not investigate effects on the immune system (77). The proportion and types of lipids in the diet influence the gut microbiota composition. In healthy C57Bl/6 mice, mice were subjected to a high fat diet containing palm oil (high in saturated fatty acids), olive oil (high in monounsaturated fatty acids), safflower oil (high in Ω-6 polyunsaturated acids), or flaxseed/fish oil (high in Ω-3 polyunsaturated fatty acids). These diets were also compared to two low-fat diets, of which one contained a high percentage of calories from maize and one diet in which most calories came from sucrose. The cecum contents were tested for microbiota composition. In the palm oil group, the relative abundance of Bacteroidetes and *Bacteroidaceae* was reduced, while *Lachnospiraceae* was increased. Mice that received an olive oil-based diet had an increased proportion of *Bacteroidaceae*. In the flaxseed/fish oil group, the proportion of *Bifidobacteriaceae* and *Bifidobacterium* was increased. Within the low-fat diets, the relative abundance of *Ruminococcaceae* was increased and *Erysipelotrichaceae* was reduced in the high sucrose diet. Interestingly, cecal concentrations of SCFA were increased in the palm oil supplemented group (104). This indicates that dietary fats greatly influence the gut microbiota composition, which influences SCFA production or absorption. In this way, shifts in gut microbiota composition as a result from dietary fats may influence EAE/MS.

Other dietary components can also modify EAE disease scores. Potentially relevant for MS is the beneficial effect of vitamin D3 on EAE, as vitamin D3 deficiency is an established MS risk factor. Intraperitoneal administration of vitamin D prevents EAE development in SJL/J mice immunized with rat spinal cord homogenate when given prophylactically (65). Additionally, dietary vitamin D supplementation reduces EAE scores when given to MBP_79–87_-immunized B10.PL mice (66) and MOG_35–55_-immunized C57Bl/6 mice with established EAE, in which the effect was IL-10 dependent (67). DC isolated from MOG_35–55_-immunized C57Bl/6 mice were also able to reduce EAE severity when incubated with 1,25-dihydroxyvitamin D3 prior to adoptive transfer into mice with EAE. Even though the proportions of T_h1_ and Th17 cells in lymph nodes and the spleen were increased during this treatment, their proportions in the spinal cord were reduced. Therefore, DC that were exposed to 1,25-dihydroxyvitamin D3 may reduce migration of pathogenic T cells from the periphery into the CNS (105). *In vitro* culture with high concentrations of vitamin D inhibits CD4^+^ T cell proliferation, reduces IL-6, IL-17 producing T-cells while enhancing IL-10 producing and CD4^+^CD25^+^Foxp3^+^ T cells (106). In other models, vitamin D reduced demyelination and increased remyelination (107) and exerted additional anti-inflammatory effects, such as inhibition of the T_h1_ and B-cell response and modulation of DC (108). Vitamin D may also inhibit the bacteria-induced pro-inflammatory NF-κB pathway and affect tight junction expression, contributing to proper intestinal barrier function (108).

Excess calorie intake and/or fasting may have a negative effect on MS/EAE. A prophylactic diet of 66% caloric restriction protected Lewis rats from developing EAE (69), while a prophylactic caloric restriction of 40% reduced the EAE score in several mouse models, which was associated with reduced spinal cord inflammation, demyelination, and axonal injury (68). Additionally, increased plasma levels of corticosterone, adiponectin and reduced plasma levels of IL-6 and leptin were found in animals with reduced food intake (68). These altered plasma concentrations are interesting, as corticosterone has broad inhibitory effects on the immune system, and adiponectin reduces IL-6 and TNFα production as well as induces production of IL-10R and IL-1R antagonists. Leptin induces T cell proliferation, T_h1_ differentiation, and pro-inflammatory cytokine production (68). Intermittent feeding (*ad libitum* access to food on alternating days) also had a positive effect on the EAE clinical score, but its effect on the immune system was not investigated (70). The beneficial effect of fasting on EAE disease score was confirmed in another study, in which mice were fed in cycles of 4 days *ad libitum*, followed by 3 days of fasting (first day of fasting 50%, then 2 days of 10% of normal caloric intake). This intervention was able to reduce the clinical EAE score, even in mice with established EAE. The suppression of EAE was associated with reduced immune cell infiltration into the spinal cord, reduced splenic CD11^+^ DC, reduced pro-inflammatory cytokines, increased corticosterone in serum, increased T_reg_, and reduced T_h1_ and Th17 cells in the lymph nodes and spleen. In addition, fasting protects oligodendrocytes from apoptosis and stimulates maturation of oligodendrocyte precursors (77). The effect on oligodendrocytes is seen in EAE and also in a cuprizone model, which demonstrates that fasting affects oligodendrocytes also in an autoimmunity-independent way (77).

Increased salt intake increased the EAE disease score in MOG_35–55_-immunized C57Bl/6 mice (78–81). This effect was associated with increased activity of Th17 cells in the spinal cord and spleen. It is thought that the effect of high sodium is DC independent, as DC function is not altered by exposure to salt (78). Instead, sodium directly influences T_h17_ differentiation. Two studies found that high sodium induces the expression of serum/glucocorticoid kinase 1 (SGK-1) in naïve T cells (80, 81). SGK-1 promotes IL-23R expression and induces T_h17_ differentiation (80). The importance of SGK-1 signaling is shown by the observation that a high-salt diet increases the severity of EAE in mice, while SGK-1-deficient mice develop less severe EAE. This effect was associated with reduced Th17 cells in the CNS and mesenteric lymph nodes in SGK-1-deficient mice (80). Dietary tryptophan can be metabolized into a variety of AHR ligands. AHR ligands have shown different effects on EAE when given prophylactically. The tryptophan-derived AHR ligand 6-formylindolo[3-2b]carbazole (FICZ) accelerates EAE onset and increases pathology in MOG_35–55_-immunized C57Bl/6 mice. This is associated with increased IL-17 and IL-22 producing CD4^+^ T cells in the spinal cords (82). Another study verified that FICZ increases EAE severity and shows that FICZ-treated mice have increased IL-17^+^CD4^+^ and IFNγ^+^CD4^+^ T cells in the spleen (83). In addition, they show that tryptophan-derived 2-(1′H-indole-3′carbonyl)-thiazole-4-carboxylic acid methyl ester (ITE) reduces EAE severity. Stimulation with 2,3,7,8-tetrachlorodibenzo-p-dioxin (TCDD), a less natural AHR ligand, induces T_reg_ cells, which are protective of EAE also after adoptive transfer (83). Even though in these studies the AHR ligands were injected rather than supplemented through diet, it shows the immunomodulatory effects of different AHR ligands which may also be taken through the diet. AHR signaling may also have protective effects through astrocytes. Mice fed a tryptophan-deficient diet develop increased EAE severity. This effect could be reversed by tryptophan supplementation in control mice, but not in astrocyte-specific AHR knockout mice. Gut microbiota mediate the conversion of tryptophan into AHR ligands. Depletion of these microbiota by ampicillin treatment increased EAE disease scores, which can be reduced by supplementation with multiple tryptophan metabolites and the bacterial enzyme tryptophanase. This shows a protective effect of the interplay between dietary tryptophan, gut microbiota, AHR ligands, and astrocytes (84).

Zinc can also influence EAE. *In vitro*, salt composed of zinc with the amino acid aspartate (zinc aspartate = ZnAsp) reduces the proliferation of stimulated human T-cells (85, 86) and also reduces their production of IL-2, IL-10, IL-17 (85), IFNγ, TNFα, GM-CSF, and IL-5 (86). The same effects of ZnAsp were seen in mouse splenocyte cultures (85, 86). In PLP_139–151_-immunized SJL mice, intraperitoneal injection of 30 µg ZnAsp per day reduced the EAE clinical score prophylactically as well as therapeutically, while 120 µg increased the EAE severity (85). An oral dose of 6 or 12 µg ZnAsp per day reduced the clinical disease score in the same EAE model, while an oral dose of 30 µg increased the disease score (86). Zinc supplementation also affected EAE in the MOG_35–55_-immunized C57Bl/6 model. Zinc supplementation in drinking water (87) and daily intraperitoneal injections of 6 and 30 µg ZnAsp both reduced the EAE clinical score when given before EAE induction (88). The mechanisms underlying the effect of ZnAsp injections include reduced systemic Th17 cells and increased Foxp3^+^ T-cells in the spinal cord (88). Lower plasma or serum concentrations of zinc have been found in MS patients compared to healthy individuals, while others have found no differences. Mice with EAE had reduced zinc plasma levels on day 21 after EAE induction compared to naïve mice (86). Given the beneficial effects of zinc supplementation in EAE, normalizing zinc levels in MS patients with zinc deficiency may be tested in clinical trials, but a high dosage or long-term supplementation may also have detrimental effects, thus patient serum concentrations in these trials must be tightly monitored (86). A zinc-deficient diet of 10 days did not alter the microbiota composition at phylum level and total bacterial abundance in healthy C57Bl/6 mice compared to those that were fed a normal diet. The relative abundance of several bacterial genera did differ between these diet groups, which included the genera *Enterococcus, Enterobacteriaceae, Paenibacillus, Granulicatella, Clostridium, Akkermansia*, and *Burkholderia* (109).

High dietary selenium (10× normal intake) increased EAE incidence and severity in guinea pigs, while those on normal and half amounts of selenium developed EAE in a normal fashion (89).

Intraperitoneal injection of diphenyl diselenide was toxic for whole MBP-immunized Wistar rats, while oral administration 1 week after immunization resulted in reduced EAE incidence and symptoms. This was associated with reduced macrophage numbers in the CNS and reduced MBP-specific T-cell proliferation. The EAE-reducing mechanism of diphenyl diselenide is thought to be based on reduced NF-κB signaling in macrophages and T-cells. In addition, diphenyl diselenide may increase ROS clearance as it mimics glutathione peroxidase activity, which protects against oxidative damage (90). The different effects of dietary selenium on EAE may be explained by differences in formulation.

Vitamin E has potent antioxidant properties. Multiple compounds are considered part of the vitamin E group, of which tocopherol is best studied. Intraperitoneal administration of tocopherol reduced the EAE clinical scores in MOG_35–55_-immunized C57Bl/6 mice, which was associated with reduced MOG-specific splenocyte proliferation. In addition, splenocytes incubated with tocopherol produced less IFNγ (91). Vitamin E also increases remyelination and reduces demyelination in an animal model where demyelination is chemically induced (107). TFA-12, a synthetic tocopherol derivative, reduced the EAE clinical scores in MOG_35–55_-immunized C57Bl/6 mice when injected intraperitoneally at the onset of EAE symptoms. This was associated with reduced inflammation of the CNS, astrogliosis, and demyelination. TFA-12 also accelerated remyelination in a chemically induced demyelination model. The mechanism behind this is believed to be due to the induction of oligodendrocyte maturation (92).

Vitamin A and its metabolites all-trans retinoic acid and 9-cis-retinoic acid can also reduce EAE severity. Prophylactic dietary 13-cis-retinoic acid and 4-HPR (a synthetic retinoid derivative) reduced EAE incidence in SJL mice which received MOG-incubated lymph node cells to induce EAE. Dietary 4-HPR can also reduce EAE severity in a therapeutic manner (93). In MOG_35–55_-immunized C57Bl/6 mice, intraperitoneal injection with all-trans retinoic acid reduced the EAE clinical score when given prophylactically (94) as well as therapeutically (95). This was associated with reduced DC maturation and reduced pro-inflammatory monocytes in the subarachnoid space. Reduced T_h1_ and Th17 cells were also found in the draining lymph nodes. In addition, bone mesenchymal DC pretreated with all-trans retinoic acid were able to reduce T_h1_ and T_h17_ differentiation and lymphocyte proliferation *in vitro* (94). All-trans retinoic acid also directly affected naïve CD4^+^ T cells, as it inhibited T_h17_ differentiation by reducing expression of the IL-6 and IL-23 receptor (95). Even though all-trans retinoic acid can induce Foxp3^+^ T cells *in vitro*, the frequency of these cells was not increased in EAE-affected mice treated with the metabolite (95). *In vitro*, 9-cis-retinoic acid reduced pro-inflammatory cytokine production in LPS-stimulated microglia and reduced TNF-α and nitric oxide production in astrocytes (110). Mechanisms underlying gut microbiota and dietary interventions are summarized in Box 2.

Box 2 **Mechanisms underlying microbiota and dietary interventions in experimental autoimmune encephalomyelitis (EAE).**

Most interventions are mediated by the induction of anti-inflammatory IL-10 producing T_regs_. These cells suppress pro-inflammatory cytokine production and T cell proliferation. In addition, multiple other mechanisms have been unraveled.

*Germfree* mice are resistant against EAE. The EAE resistance is attributed to reduced recruitment and activation of autoantibody producing B cells (47) as well as dendritic cells (DC) and the reduced capacity of these professional antigen-presenting cells (APC) to stimulate pro-inflammatory T cell responses (48).

*Antibiotic* treatments reduce EAE *via* invariant natural killer T (iNKT) cells and CD1^high^CD5^+^ B cells. Antibiotic treatments likely suppress pro-inflammatory cytokine production by iNKT cells located in the lamina propria, which reduces T_h17_ development (49). Antibiotic treatment also induces IL-10 producing CD1^high^CD5^+^ B cells in distant lymph nodes, and adoptive transfer of CD1^high^CD5^+^ B cells protect against EAE (51). Antibiotic treatments increase CD11c^high^CD103^+^ DC in mesenteric lymph nodes, which are able to induce Foxp3^+^ T cells (50).

*Polysaccharide A (PSA)* is captured by DC in the gut *via* a TLR-2 dependent mechanism. DC then migrate to the mesenteric lymph nodes and induce IL-10 producing CD4^+^ T_regs_. A type of IL-10 producing T_reg_ specifically induced by PSA expresses the ectonucleosidase CD39 on its surface. CD39^+^ T_reg_ cells have increased migratory capacity, are more abundant in the central nervous system (CNS) of PSA-treated mice, and are protective against EAE. The EAE modulatory capacity relies on the conversion of ATP into adenosine, which increases the anti-inflammatory effects of T_regs_ and suppresses effector cells, thus protecting against CNS inflammatory tissue damage (61, 99). Additionally, PSA treatment causes accumulation of CD103^+^ DC in the cervical lymph nodes, which converses naïve CD4^+^ T cells into protective IL-10 producing Foxp3^+^ T cells. The mechanism causing the accumulation of CD103^+^ DC in the cervical lymph nodes is unknown (59).

*Short-chain fatty acids (SCFA)*, such as butyrate and propionic acid, are produced by bacterial fermentation of dietary fibers in the colon or consumed with the diet. SCFA induce T_regs_ through multiple known mechanisms. Oral administration of propionic acid can induce CD4^+^CD25^+^Foxp3^+^ T_reg_ cells, while simultaneously reducing T_h17_ responses (64). Butyrate can stimulate DC, macrophages, and epithelial cells to produce cytokines that facilitate the differentiation of naïve CD4^+^ T cells into anti-inflammatory Foxp3^+^ T cells (100). *Dietary components* influencing EAE also include: (1) Different types and ratios of lipids, such as Ω-6 fatty acids, Ω-3 fatty acids and ketogenic diets reduce EAE by affecting splenic mononuclear cells (71) or T cells (73). (2) *Vitamin D3* has a broad effect on the immune system (108). In EAE, its effect is dependent on IL-10 (67). (3) *Vitamin E* has antioxidant properties (91), accelerates remyelination (107), and also affects splenocytes (91). (4) *Vitamin A* affects DC (94) and reduces T_h1_ and T_h17_ differentiation (95). (5) *Zinc* increases Foxp3^+^ T cells (88), reduces T cell proliferation, and reduces their pro-inflammatory cytokine production (85, 86). (6) *Selenium* protects against oxidative damage and also affects T cells (90). (7) *Caloric restriction/fasting* reduces EAE, which is associated with changes in plasma levels of corticosterone, adiponectin, IL-6, and leptin (68). In addition, fasting reduces DC, T_h1_, Th17 cells and increases T_reg_ cells as well as stimulates remyelination by oligodendrocytes (77). (8) A *high-salt* diet increases the T_h17_ response, which is likely DC independent. Instead, salt induces SGK-1 signaling, leading to increased T_h17_ differentiation. (9) *Aryl hydrocarbon receptor ligands* can have multiple effects on EAE. Cruciferous vegetables such as broccoli are rich in indole-3-carbinol, which upon contact with stomach acid, is converted into aryl hydrocarbon receptor ligands and can induce CD4^+^ IEL. These cells can migrate into the CNS and are protective against EAE (62). 6-Formylindolo[3-2b]carbazole, a different aryl hydrocarbon receptor (AHR) ligand, increases the EAE disease score, while the tryptophan-derived AHR ligand ITE reduces EAE clinical signs. Tryptophan-derived AHR ligands limit astrocyte-mediated inflammation in the CNS (84). Aryl hydrocarbon receptor ligands may also act on type 3 ILC (17).

## Gut Microbiota in MS Patients

### Comparing the Gut Microbiota of MS Patients with Healthy Controls

Colonization with bacteria and oral treatment with bacterial products and diet are all exogenous factors *via* which the clinical and/or pathological expression of EAE can be modified. It is therefore of considerable interest whether differences exist between the gut microbiota composition of MS patients compared to healthy individuals and to test whether treatment concepts developed in EAE can be translated to humans. Studies analyzing gut microbiota of MS patients are summarized in Table 3. Of these studies, eight have investigated whether differences exist in the microbiota composition of MS patients compared to healthy individuals, for which collectively almost 250 MS samples were tested. A reduced abundance of SCFA producers such as *Lachnospiraceae, Prevotella, Faecalibacterium prausnitzii*, and *Butyricimonas* in the gut of MS patients has been repeatedly reported. As SCFA reduced the disease score in several EAE models, reduced SCFA production in the gut of MS patients may have an impact on MS pathology. Of note, *F. prausnitzii* produces a microbial anti-inflammatory molecule (MAM), which has anti-inflammatory effects in a mouse model of colitis. MAM displays an inhibitory effect on nuclear factor-κB activity in human epithelial cells. Moreover, administration of *L. lactis* delivering a MAM-encoding plasmid reduced morbidity in an animal colitis model, whereas wild-type *L. lactis* had no effect (111). By producing MAM, *F. prausnitzii* adds to an anti-inflammatory milieu in the gut. Additionally, the gut of MS patients harbors higher numbers of the archaea *Methanobrevibacter* compared to healthy individuals. As mentioned earlier, lipids from *Methanobrevibacter* are thought to elicit inflammatory reactions (112) and therefore possibly add to a pro-inflammatory milieu in the gut of MS patients. Two studies found that *Enterobacteriaceae* are increased in MS patients compared to healthy individuals. The *Enterobacteriaceae* family of gut bacteria includes pathogenic as well as non-pathogenic/opportunistic species. Increased *Enterobacteriaceae* abundance has been found in patients with inflammatory bowel disease (IBD). However, it is unclear if this dysbiosis is a cause or consequence of the IBD (3). Several *Enterobacteriaceae* members have a competitive growth advantage under inflammatory conditions in the gut of mice (113), and therefore, it is conceivable that *Enterobacteriaceae* abundance increases after IBD develops. Studies investigating the role of *Enterobacteriaceae* in EAE models were not found. Of note, children with autism and patients with Parkinson’s disease have increased *Enterobacteriaceae* abundance in their gut microbiota, indicating that *Enterobacteriaceae* could possibly play a role in CNS disorders (114). Only one study found that *B. fragilis* was reduced in a cohort of 18 pediatric MS patients compared to healthy individuals (115). *In vitro*, PSA induced expression of CD39 and Foxp3 on naïve human CD4^+^ T cells and increased IL-10 production (116). Even though only one study found a difference in the abundance of *B. fragilis* (115), the beneficial effect of PSA on EAE may be translatable to corresponding human diseases, such as MS.

**Table 3:** Gut microbiota studies in MS patients.

Reference	Number of subjects, type of MS	Main findings
Tremlett et al. (115)	18 RR pediatric, 17 HC	No difference in species richness. Increased *Desulfovibrionaceae, Methanobrevibacter*^b^, *Enterobacteriaceae*^b^. Reduced *Lachnospiraceae*^a^, *Ruminococcaceae*^a^, *Faecalibacterium prausnitzii*^d^, and *Butyricimonas*^a^ *Bacteroides fragilis*^c^. Functional prediction of microbial genes indicated increased glutathione metabolism in patients

Tremlett et al. (117)	17 RR pediatric	Depletion of Fusobacteria is associated with increased risk on earlier relapses. Higher abundance of Firmicutes and Euryarchaeota trended to be associated with increased risk on earlier relapses

Tremlett et al. (118)	15 RR pediatric, 9 HC	No difference in blood Foxp3^+^ T_reg_ frequency and intracellular production of IFNγ, IL-17, IL-4, and IL-10 by CD4^+^ T cells. IL-17^+^ T cells correlated with gut microbiota richness in MS patients. IL-17^+^ T cells inversely correlated with Bacteroidetes abundance in patients. T_reg_ frequency correlated with Fusobacteria abundance in healthy controls

Miyake et al. (119)	20 RR, 40 HC, 18 HC	No difference in species richness. Reduced *Faecalibacterium*^d^, *Prevotella*^a^, *Anaerostipes*, butyrate-producing bacterium A2-175^a^, and SL7/1^a^. Trends in increased *Bifidobacterium, Streptococcus*

Chen et al. (120)	31 RR, 36 HC	No difference in species richness. Increased *Pedobacter, Flavobacterium, Pseudomonas, Mycoplana, Blautia, Dorea*, and *Haemophilus*. Reduced *Parabacteroides, Adlercreutzia, Collinsella, Lactobacillus*, and *Prevotella*^a^. Reduced fatty acids metabolism and increased phytoestrogen metabolism were predicted by functional analysis

Jangi et al. (112)	60 RR, 43 HC	Increased *Methanobrevibacter*^b^, *Akkermansia*. Decreased *Butyricimonas*^a^. Untreated patients had reduced *Prevotella*^a^, *Sutterella*, increased *Sarcina*. Positive correlations between *Methanobrevibacter*^b^, *Akkermansia*, and T-cell/monocyte gene expression implicated in MS pathology. Negative correlations for *Butyricimonas*^a^ with gene expressions implicated in MS pathology

Jangi, et al., abstract (121)	22 untreated, 13 GA, 18 IFN-β, 44 HC	Increased *Methanobrevibacteriaceae*^b^ in patients compared to HC. Reduced *Butyricimonas*^a^ in untreated patients compared to HC. Both treatment groups had increased *Lachnospiraceae*^a^ abundance compared to untreated MS patients

Baum et al., abstract (122)	54 MS patients vs healthy controls (the amount of controls is not stated in the abstract)	Increased *Atopobium, Bifidobacteriae*. Reduced *Bacteroidaceae* in MS patients

Cantarel et al. (123)	7 RR vitamin D-deficient, before and after vitamin D supplementation, 8 HC	Reduced *Bacteroidaceae, Faecalibacterium*^d^, increased *Ruminococcus*^a^ in patients. Vitamin D supplementation increased *Faecalibacterium*^d^, *Akkermansia*, and C*oprococcus*^a^. GA treatment affected gut microbiota

Sand et al., abstract (124)	Not specified in abstract	Increased *Enterobacteriaceae*^b^ in female patients compared to HC. GA treatment affects gut microbiota

Tankou et al., abstract (125)	43 untreated MS patients, disease subtype not specified	Patients with <40 ng/mL serum vitamin D concentration had lower *Ruminococcaceae*^a^ compared to patients with higher vitamin D levels

Telesford et al. (116)	*In vitro* cultures of patients and HC	PSA from *B. fragilis*^c^ induced CD39^+^ T_reg_ cells *in vitro*

*^a^SCFA producer*.

*^b^Likely pro-inflammatory*.

*^c^PSA producer*.

*^d^MAM producer*.

*MAM, microbial anti-inflammatory molecule; MS, multiple sclerosis; PSA, polysaccharide A; RR, relapsing remitting; SCFA, short-chain fatty acids*.

**Figure 5:**
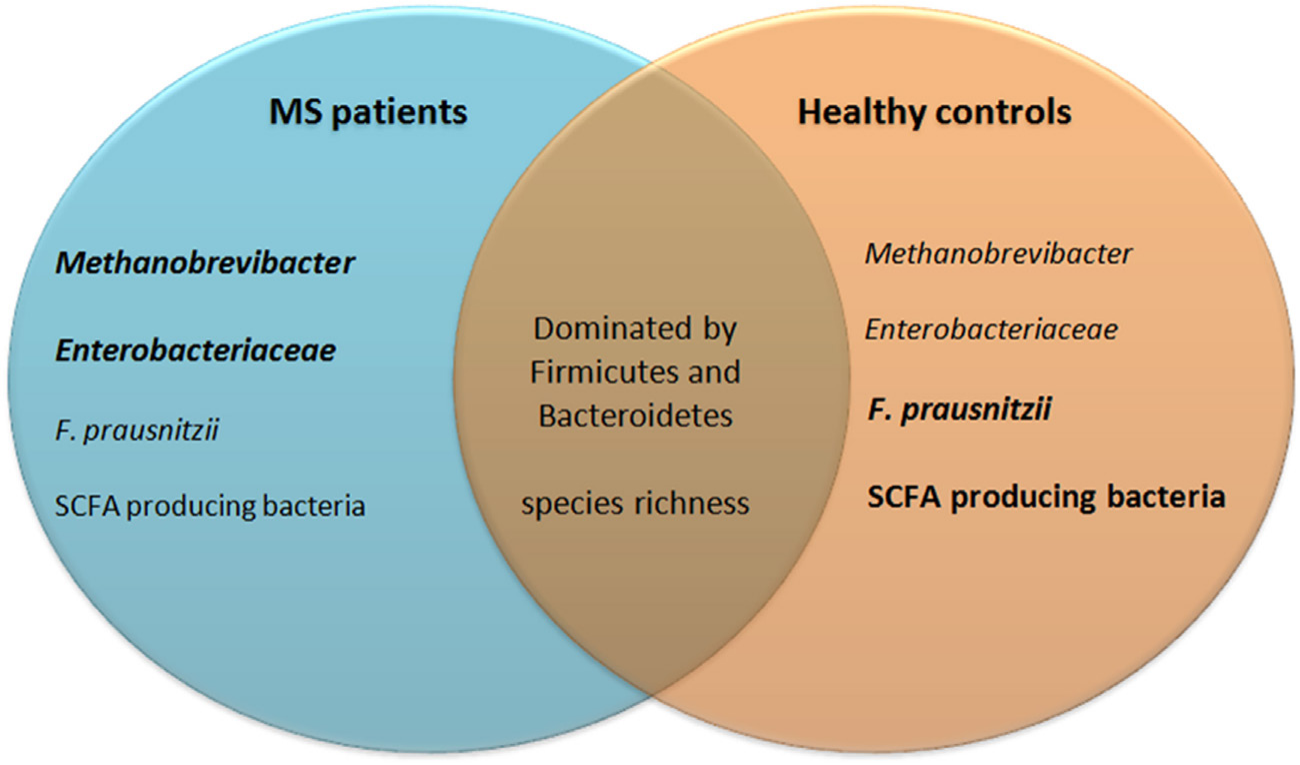
Similarities and differences in gut microbiota of MS patients and healthy controls. Eight studies investigating almost 250 patient fecal samples for differences in microbiota composition were analyzed. Many species were shown differently present. Only differences that have been reproduced by at least one other study are included in this figure. The gut microbiota of both MS patients and healthy controls are dominated by bacteria from the phyla *Firmicutes* and *Bacteroidetes* and their species richness does not differ. MS patients may have increased *Methanobrevibacter* and *Enterobacteriaceae*, but reduced *Faecalibacterium prausnitzii* and SCFA producing bacteria. SCFA, short-chain fatty acids; MS, multiple sclerosis.

Taken together, the gut microbiota composition of MS patients and healthy individuals likely differs, as reflected by a different abundance of SCFA producing bacteria, *F. prausnitzii, Methanobrevibacter*, and *Enterobacteriaceae* (see Figure 5). Not all studies reported the same differences between MS patients and healthy individuals. This may be explained by differences in sampling methods, molecular techniques, and differences in study populations such as their diet, received treatments, type of MS, or ethnicity. In addition, the populations of tested MS patients varied among studies. Differences in the gut microbiota of pediatric MS patients compared to adult MS patients may exist. Moreover, some studies divided patients in groups based on treatments, while others only separated patients from healthy individuals. Therefore, there is a great need of large controlled studies to determine which results from multiple small studies can be replicated in a bigger cohort. It is also not clear whether the dysbiosis in SCFA-producing bacteria, *F. prausnitzii, Methanobrevibacter*, and *Enterobacteriaceae* adds to the pathology of MS, or if it is a result of MS pathology or treatments.

### Prospective Microbiota Studies in MS Patients

Two relatively small studies have tried to link gut microbiota to clinical outcomes or changes in the immune system of MS patients. In a cohort of 17 pediatric MS patients, the absence of *Fusobacteria* was associated with increased risk on earlier relapses. Additionally, trends in increased abundance of *Firmicutes* and *Euryarchaeota* were observed which may be associated with increased risk of earlier relapses (117). In 15 pediatric MS patients, no difference was found in blood CD4^+^ T_reg_ cells compared to healthy individuals, but IL-17^+^ T cells correlated with species richness and inversely correlated with *Bacteroidetes* in patient stools (118). These studies are an interesting indication that it may be possible to modulate the gut microbiota as a treatment of MS. However, studies performed thus far included far too few patients to be able to determine a causal relationship between the abundance of certain microbial species and symptoms or immune responses. The effect of MS treatments on gut microbiota has also only been investigated in relatively small studies thus far. Preliminary data suggest that both glatiramer acetate treatment and vitamin D supplementation affect gut microbiota (see Table 3).

The aforementioned microbiota studies in MS patients are possibly biased as fecal/stool samples were used for characterization of gut microbiota. The composition of microbiota varies from the mucosal surface to the lumen, and fecal samples mostly detect luminal microbiota. Hence, differences in mucosa-associated species may be underappreciated (17). *B. fragilis* is one of the few species that can penetrate the tight inner mucus layer of the colon and can colonize the colonic crypts (15). It is, therefore, interesting to investigate whether mucosa-associated *B. fragilis* is differently present in patients. Moreover, as fecal samples more closely reflect microbiota composition of the colon than of the small intestines, differences in microbiota composition of the small intestines are undetected (16).

### Potential Role of PGN in MS

Another way in which gut microbiota may influence MS is through PGN, a bacterial cell wall component. PGN is ubiquitously present in the gut and can be phagocytosed into APC (142). PGN is sensed through NOD-like and toll-like receptors, which induces pro-inflammatory mitogen-activated protein kinase and NF-κB (143). Microbiota-dependent NOD1 signaling increases the lifespan of circulating neutrophils and monocytes (144), and PGN has strong adjuvant properties, as mice injected with MOG_35–55_ and incomplete Freund’s adjuvant did not develop EAE, while when PGN was added to the mixture mice did develop EAE (145). Mechanistically, PGN modulates DC, which leads to Th1 cell expansion (145). In the rhesus monkey and marmoset EAE model (146) as well as human MS (147), PGN is found in APC located in the brain. Another study in autopsied brain samples revealed that the expression of bacterial components in MS samples differed from non-MS samples. In both sample groups, the dominant phylum was Proteobacteria, but less diversity was observed in progressive MS samples. In addition, Actinobacteria were enriched in RRMS samples. The total amount of PGN in the brain did not distinguish MS samples from non-MS samples. But within MS lesions, PGN inversely correlated with myelin density and was associated with several genes of the immune system, including NF-κB (34). The presence of a TLR/NLR ligand in the brain, possibly originating from gut microbiota, may contribute to MS pathology by influencing inflammation, demyelination or remyelination, and therefore, more research is needed to elucidate the role of microbial compounds in MS brains.

## Dietary Studies in MS Patients

How different food components may affect MS has been reviewed comprehensively by Schmitz et al. (141) of which the most important ones are summarized in Table 4. Multiple dietary intervention studies have been done in MS patients, but most were unsuccessful in reducing MS severity and have not examined the effects on microbiota. Vitamin D supplementation did show positive outcomes as it led to fewer relapses and reduced pro-inflammatory cytokines such as IFNγ and IL-4 in T-cells. Additionally, vitamin D supplementation increased peripheral IL-10^+^CD4^+^ T cells, suppressed T-cell proliferation, and reduced the number of gadolinium-enhancing regions per patient (13, 141). However, these results came from small studies and more randomized controlled trials investigating the effect of vitamin D for MS treatment are now taking place. The effect of vitamin D might in part be exerted *via* the gut microbiota, as in healthy individuals high vitamin D intake was associated with increased proportions of *Prevotella* and reduced proportions of *Haemophilus* and *Veillonella* (148). Vitamin D supplementation in vitamin D-deficient MS patients altered the relative proportions of different genera, but this study included very few subjects (123). In a study of 70 and a replication study of 59 RR MS patients, the influence of salt on MS was studied. As 80–90% of salt intake is excreted in urine, salt excretion rate in urine was measured as a proxy for salt intake. High salt excretion was associated with increased relapse rates, increased risk on developing a new lesion detected by MRI scans, and increased T2 lesion load. However, causality was not established. Patients with relapses are often treated with steroids, which influences salt excretion (137). Clinical trials with controlled salt intake are needed to assess the impact of increased salt intake on MS severity.

**Table 4:** Dietary studies in MS patients.

Reference	Number of subjects, type of MS	Diet-groups	Main findings
Bates et al. (126)	292 RRMS	Ω-3 EPA and DHA vs oleic acid supplementation. Both groups also had vitamin E and antioxidant supplementation	No difference in relapse rate or EDSS

Weinstock-Guttman et al. (127)	27 RRMS	Low-fat diet (<15% calories) with Ω-3 EPA and DHA supplements vs low fat (<30% calories) with oleic acid supplements. Both groups received vitamin E, multivitamin, and calcium supplementation	No difference in relapse rate or EDSS between groups. Relapse rates, reduced compared to 1 year before the start of this study. EPA/DHA group had increased physical and mental parameters. Reduced fatigue score in the oleic acid group

Torkildsen et al. (128)	99 RRMS	Ω-3 EPA and DHA vs corn oil supplementation	No difference in relapse rate, EDSS, quality of life, and fatigue scores

Bates et al. (129)	134 SPMS	Ω-6 Linoleic acid and γ-linolenic acid vs linoleic acid vs oleic acid supplementation	No difference in relapse rate or EDSS

Bates et al. (130)	104 PPMS	Ω-6 Linoleic acid and γ-linolenic acid vs linoleic acid vs oleic acid supplementation	No difference in relapse rate or EDSS. High-dose linoleic acid group had less severe relapses

Harbige and Sharief (131)	28 RRMS	Ω-6 Linoleic acid supplementation vs placebo	Reduced relapse rate, improved EDSS

Jafarirad et al. (132)	35 RRMS	Vitamin A (retinyl palmitate) or placebo supplementation	Reduced T cell proliferation when incubated with MOG

Wingerchuk et al. (133)	15 RRMS	Vitamin D supplementation, uncontrolled	Reduced EDSS compared to baseline

Mahon et al. (134)	39 MS patients, subtype not specified	Vitamin D3 supplementation vs placebo. Both groups received calcium supplementation	Increased TGF-β concentration in serum of vitamin D3 supplemented group. No differences in TNFα, IFNγ, and IL-13 concentrations

Goldberg et al. (135)	10 MS patients, subtype not specified	Vitamin D3, calcium, and magnesium supplementation, uncontrolled	Fewer relapses than expected

Choi et al. (77)	48 RRMS	Cycles of fasting vs ketogenic diet vs control	Fasting and diet group had improved health related quality of life, reduced disability scores. Fasting and ketogenic diet were well tolerated

Haghikia et al. (136)	Not specified in abstract	Propionic acid treatment in patients and HC	No side effects of PA. 25–30% increase of T_reg_ and reduced Th17 cells in both groups

Farez et al. (137)	70 RRMS, replicated by a separate group of 52 patients		High salt excretion is associated with increased disease activity

Hadgkiss et al. (138)	2,087 MS patients, subtype not specified		A general healthy diet, based on fruit, vegetable, fat, meat and dairy consumption was associated with better clinical scores

Rezapour-Firouzi et al. (139)	65 RRMS	Three groups: 1. hemp seed/evening primrose oil; 2. olive oil; and 3. cosupplemented oil vs baseline measurements. Subjects were advised to have a general healthy diet	Reduced relapse rates, EDSS in groups 1 and 3 compared to baseline

Nordvik et al. (140)	16 RRMS	General health lifestyle and A, B, D, E, Ω-3 fatty acid supplementation	Reduced EDSS compared to baseline

*Dietary studies in MS patients have been extensively reviewed by Schmitz et al. (141)*.

*AHR, aryl hydrocarbon receptor; CNS, central nervous system; DC, dendritic cells; DHA, docosahexaenoic acid; EDSS, expanded disability status scale; EAE, experimental autoimmune encephalomyelitis; EPA, eicosapentaenoic acid; MOG, myelin oligodendrocyte glycoprotein; MS, multiple sclerosis; SGK-1, serum/glucocorticoid kinase 1*.

In a cohort of 2,087 MS patients, a general healthy diet (based on fruit/vegetable, fat, meat, and dairy consumption) was associated with better clinical scores (138). Therapeutically, two studies have shown effect of a general healthy diet (low intake of saturated fatty acid, sugar, coffee, alcohol, and high intake of vegetables, fruits, fish and whole grain products) on MS clinical scores when combined with supplementation of oils rich in Ω-6 or Ω-3 fatty acids (139). Of note, one of these studies also supplemented multiple vitamins including vitamin A, B, D, and E (140). Since these studies used multiple dietary components as intervention, it is hard to determine which part of the intervention exerted the beneficial effects. A difference in fat consumption may be responsible, since a third study found that reduced fat intake supplemented with Ω-3 fatty acids reduces MS clinical scores (127). It is noteworthy that the abovementioned studies related results to baseline measurements instead of a control group. Because dietary studies in MS can hardly be blinded and patients in the control groups have little incentive to complete a study, control groups have high dropout rates. Efforts are now being made to develop study designs that increase the participation of patients in control groups and to test if an observation period prior to a test diet can serve as a control group (149). Multiple studies are currently listed on www.clinicaltrials.gov as recruiting or ongoing, including studies testing diets such as ketogenic, low sodium, low saturated fat, caloric restriction, and a variation of Paleolithic diet (low grain, high vegetables). These studies should provide additional insight into which compounds may be responsible for the positive effects of a generally healthy diet. Preliminary results of oral treatment with propionic acid are promising. Healthy individuals and patients were treated with propionic acid, which increased T_reg_ frequency and reduced T_h17_ frequency in both groups without any side effects; only an abstract of this study is available (136).

## Methods to Manipulate Gut Microbiota for Therapeutic Purposes

Current evidence suggests that MS patients may have a different gut microbiota composition compared to healthy individuals and that gut microbiota may affect the relapse rate (see Table 3). If these results will be verified in larger studies, it would be interesting to test whether manipulation of the gut microbiota of MS patients induces immunosuppression. Multiple approaches can be used to manipulate the gut microbiota (see Box 3). Probiotics involve the treatment with live microorganisms that are absent or present in too low abundance in the host. In this way, bacteria that induce immunosuppression can be introduced in patients. Probiotics are already being used in intestinal diseases such as IBD or antibiotic-associated diarrhea (17). Prebiotics are food ingredients with which gut microbiota may be modified. Resistant starches, which can reach the colon before being metabolized, alter the gut microbiota and have been associated with increased butyrate production. Additionally, fibers can be fermented by gut microbiota that produce SCFA. However, the effect of prebiotics depends on microbial strains that are already present in the host. Therefore, high individual variation and a large number of non-responders are seen (150). Probiotics and prebiotics can also be used in conjunction, i.e., as synbiotic, to augment their individual effectiveness. The probiotics part introduces new strains, while the prebiotic part synergistically increases the efficacy of the introduced species due to increased survival, competition, or metabolic activity (150). Antibiotics can be used to remove or suppress unwanted, immunogenic strains from the gut microbiota. A downside of antibiotic treatment is that multiple strains are targeted and beneficial bacteria may also be depleted. This increases the risk of opportunistic infections. Moreover, in mice, vancomycin treatment disrupts complex carbohydrate fermentation and leads to reduced concentrations of SCFA, including butyrate and propionic acid (151). Phages may be employed for more specific targeting of immunogenic parts of the gut microbiota. In this way, beneficial bacteria remain untouched and immunosuppression may still occur. Phages may also be used to increase the competitiveness of strains that need to be introduced. However, phage therapy still requires more research before it can be implemented, as there is limited evidence of efficacy in human clinical trials (152). Fecal microbiota transplantation involves the administration of a liquid filtrate of a healthy person’s stool to a diseased recipient. In this way, the recipient gut microbiota can be modulated to resemble the gut microbiota of the donor. New strains may be introduced by the transplantation, and through competition and possibly cross talk between gut flora and the immune system, excessive strains may be reduced and dysbiosis could be normalized. Fecal microbiota transplantation has been effective in small randomized controlled trials for the treatment of recurrent *Clostridium difficile* infection. Based on these successes, clinical trials are now being done to investigate the efficacy of fecal microbiota transplantation in IBDs (153). Fecal microbiota transplantation may be a relatively safe option for restoration of a healthy microbiome in MS patients. Instead of manipulating microbiota, microbial products that induce immunosuppression may also be used for therapeutic purposes. Chemical alterations may have to be made to make sure that the compounds reach their site of action and act through their intended mechanism. SCFA are produced by bacteria in the colon, but when orally administered, a large proportion of SCFA are thought to be absorbed in the small intestine (100). Therefore, dosage and kinetic studies will have to point out how optimal use can be made of these products. The advantage of bacterial compounds over live bacterial species is that bacterial compounds can be dosed like any other drug, while live bacterial species are affected by colonization and may therefore lead to variable interindividual effects (37).

Box 3 **Methods to alter or use microbiota as a therapeutic intervention.**

*Probiotics* can be used to introduce strains that are missing in hosts. Since multiple sclerosis (MS) patients have reduced butyrate producing strains in their gut flora, these strains may be restored using probiotics.

*Prebiotics* can be used to enrich strains that are deficiently present in the hosts. Resistant starches have been associated with increased butyrate production. Additionally, fibers can be fermented by gut microbiota which produce SCFA. The effect of prebiotics is dependent on microbial strains that are already present in the host. Therefore, high individual variation and a large number of non-responders are seen (150).

*Synbiotics* contain a mixture of probiotics and prebiotics. This mixture introduces new strains while synergistically increasing their effectiveness due to increased survival, competition, or metabolic activity of the introduced strain (150).

*Antibiotics* can be used to reduce unwanted strains in the gut microbiota. However, the effect of antibiotics is often very broad and targeting many strains at once also depletes beneficial bacteria, including short-chain fatty acids (SCFA)-producing bacteria (151).

*Phage therapy* has the key advantage that it can be used to selectively reduce strains of bacteria, but is not yet in clinical use (152). In the future, phage therapy may specifically target pro-inflammatory bacteria that are increased in MS patients, while beneficial strains remain untouched.

*Fecal microbiota transplantation* can be used in patients who have a dysbiosis to make their gut microbiota composition more closely resemble the composition of a healthy donor. Strains that are deficient in patients with a dysbiosis can be induced in the recipient using fecal microbiota transplantation, while excessive strains may be reduced through competition and possibly cross talk between gut flora and the immune system (153).

*Microbial products* such as polysaccharide A (PSA) or SCFA may be administered to induce an anti-inflammatory response in the gut. Chemical alterations may be needed to promote therapeutic efficacy and dosage/kinetic studies will need to elucidate how to best use these products (100).

*Dietary* interventions such as SCFA, Ω-3/6 fatty acid, vitamin, or trace element supplementation, reduced salt intake or caloric restriction may also be used to reduce MS.

## Conclusion and Future Directions

This review shows that many different microbial species and dietary interventions affect EAE disease expression and differences in gut microbiota between MS patients and healthy individuals may exist. Therefore, altering the gut microbiota of MS patients, possibly through dietary interventions is a potential therapeutic aim. Thus far, multiple small studies found that SCFA producing bacteria are reduced in patients with MS, and the PSA producing bacterium *B. fragilis* may also be reduced although *B. fragilis* is only found differently present in the gut by one study. Potentially pro-inflammatory *Methanobrevibacter* and *Enterobacteriaceae* may be increased in patients with MS. In addition, small studies have tried to determine if a causal relationship exists between gut microbiota and MS severity or immune responses. To determine if the results found in small studies are true and to determine if a causal relationship exists, controlled, prospective studies are eagerly awaited. Because it is relatively easy to frequently collect stool samples, it is feasible to analyze a few 100 samples of MS patients. Of these patients, clinical data need to be gathered, such as which treatments they are on, when they have relapses, as well as immunological data such as the frequency of T cell subsets and their cytokine production. In this way, the effects of vitamin D, glatiramer acetate, and other treatments on gut microbiota can be assessed, and it will be possible to determine whether a causal relationship exists between microbiota of MS patients and their immune response in relation to disease progression.

We also propose to collect mucosa biopsies to investigate the contribution of mucosa-associated bacterial species in MS. Thus far, all studies on gut microbiota in MS patients used fecal samples, which focus on luminal microbiota. Therefore, differences in mucosa-associated species may have been left undetected. Comparing intestinal biopsies of MS patients with healthy individuals may show differences, which could be additional targets for treatment. Geva-Zatorsky et al. have recently monocolonized gnotobiotic mice with one of 53 different bacterial species and assessed their immunomodulatory effects. Even though the effects of bacteria may be different in SPF or conventionally housed mice, this does provide additional immunosuppressive species which may be combined to rationally develop probiotic mixtures (37).

In the case that species that worsen or improve MS will be identified, the gut microbiota of MS patients may be modified for therapeutic benefit. This may be achieved by using probiotics, prebiotics, or a mixture of these two: synbiotics. The role of viruses in this process needs deeper investigation. As viruses can greatly influence the bacterial diversity, phage therapy may be used in conjunction with pre-, pro-, or symbiotic mixtures to reduce competitiveness of microbiota that are already present and increase colonization of introduced microbiota. These may also be used to reduce the abundance of unwanted microbial species. An already used and relatively safe option for altering the gut microbiota is fecal microbiota transplantation. The gut microbiota of MS patients may be restored with fecal microbiota transplantation to resemble the gut microbiota of healthy individuals.

An alternative for restoration of the gut microbiota of patients is treatment with bacterial products or dietary components that may dampen immune responses, such as purified PSA or SCFA. PSA is effective in EAE models through well-known mechanisms (99), and *in vitro* studies indicate that these results may translate well into humans (116). Therefore, it would be interesting to investigate if PSA would be equally effective in MS patients. Preliminary results of treatment with the dietary component propionic acid in MS patients are promising, as it shows immunological efficacy without safety concerns (136). This makes it a very promising therapeutic that needs to be tested in controlled clinical trials. The efficacy of vitamin D supplementation and reduced salt intake will also need to be tested in large randomized controlled trials.

In conclusion, gut microbiota and dietary interventions are promising treatments for MS, but there are still many questions that need to be investigated before we can conclude which bacterial species and which dietary components play a role in MS pathogenesis and before it will be possible to selectively target detrimental species as a treatment in MS patients.

## Author Contributions

All the authors developed tables, boxes, and figures. WH prepared and revised the manuscript. BH wrote part of the EAE/MS paragraphs. JL and BH critically reviewed the different versions of the manuscript and provided comments and additional research articles.

## Conflict of Interest Statement

The authors declare that the research was conducted in the absence of any commercial or financial relationships that could be construed as a potential conflict of interest.

## Abbreviations

ADCC, antibody-dependent cell-mediated cytotoxicity; AHR, aryl hydrocarbon receptor; APC, antigen presenting cells; ATP, adenosine 5′-triphosphate; CDC, complement-dependent cytotoxicity; CFA, complete Freund’s adjuvant; CNS, central nervous system; CRISPR, clustered regularly interspaced short palindromic repeats; DHA, docosahexaenoic acid; EAE, experimental autoimmune encephalomyelitis; EDSS, expanded disability status scale; EPA, eicosapentaenoic acid; FDC, follicular dendritic cells; FICZ, 6-formylindolo[3-2b]carbazole; GALT, gut-associated lymphoid tissue; GF, germfree; IBD, inflammatory bowel disease; IEL, intraepithelial lymphocytes; ILC, innate lymphoid cells; iNKT, invariant natural killer T cells; ITE 2-(1′H-indole-3′carbonyl)-thiazole-4-carboxylic acid methyl ester; KCV, kanamycin, colistin, and vancomycin; LCFA, long-chain fatty acids; MAIT, mucosa-associated invariant T; MAM, microbial anti-inflammatory molecule; MBP, myelin basic protein; MOG, myelin oligodendrocyte glycoprotein; MRI, magnetic resonance imaging; MS, multiple sclerosis; ODC, oligodendrocyte; PGN, peptidoglycan; PLP, proteolipid protein; PP, primary progressive; PSA, polysaccharide A; RR, relapsing remitting; SCFA, short-chain fatty acids; SFB, segmented filamentous bacteria; SGK-1, serum/glucocorticoid kinase 1; SP, secondary progressive; SPF, specific pathogen-free; TCDD, 2,3,7,8-tetrachlorodibenzo-p-dioxin; TNF, tumor necrosis factor.
